# Decreased SynMuv B gene activity in response to viral infection leads to activation of the antiviral RNAi pathway in *C*. *elegans*

**DOI:** 10.1371/journal.pbio.3002748

**Published:** 2025-01-29

**Authors:** Ashwin Seetharaman, Himani Galagali, Elizabeth Linarte, Mona H. X. Liu, Jennifer D. Cohen, Kashish Chetal, Ruslan Sadreyev, Alex J. Tate, Taiowa A. Montgomery, Gary Ruvkun

**Affiliations:** 1 Department of Molecular Biology, Massachusetts General Hospital, Boston, Massachusetts, United States of America; 2 Department of Genetics, Harvard Medical School, Boston, Massachusetts, United States of America; 3 Department of Biomedical and Nutritional Sciences, University of Massachusetts, Lowell, Massachusetts, United States of America; 4 Department of Biology, Massachusetts Institute of Technology, Cambridge, Massachusetts, United States of America; 5 Department of Microbiology, Harvard Medical School, Boston, Massachusetts, United States of America; 6 Department of Pathology, Massachusetts General Hospital and Harvard Medical School, Boston, Massachusetts, United States of America; 7 Department of Biology, Colorado State University, Fort Collins, Colorado, United States of America; University of Wisconsin-Madison, UNITED STATES OF AMERICA

## Abstract

RNA interference (RNAi) mediates antiviral defense in many eukaryotes. *Caenorhabditis elegans* mutants that disable RNAi are more sensitive to viral infection. Many mutants that enhance RNAi have also been identified; these mutations may reveal genes that are normally down-regulated in antiviral defense. About one-third of the score of mutants that enhance RNAi are in synMuv B genes, identified 30 years ago in unrelated screens for increased growth factor signaling. Many synMuv B genes encode dREAM complex chromatin-regulatory proteins found in nearly all animals and plants. We show that mRNAs which are highly induced in synMuv B mutants are congruent with those induced by Orsay RNA virus infection, suggesting that the enhanced RNAi of synMuv B mutants may also be triggered by down-regulation of synMuvB gene activity in an Orsay virus infection of wild type. The multivulval (Muv) phenotype of synMuv B mutants requires the presence of a second nematode-specific synMuv A gene mutation, but the enhanced RNAi of synMuv B mutants does not require a second synMuv A mutation. To test if Orsay viral infection down-regulates synMuv B gene activity, we infected a single synMuv A mutant with Orsay virus and found that a Muv phenotype could be induced. Thus, decreased synMuv B gene activity is part of the normal *C*. *elegans* viral defense response. In support of the model that decreased syn- Muv B gene activity enhances antiviral response, we found that synMuv B mutants have 50 to 100× lower viral RNA levels during an Orsay virus infection than wild type. Thus down-regulation of synMuv B activity to enhance RNAi is a key component in the defense response to viral infection. Small RNA deep sequencing analysis of dREAM complex mutants revealed siRNA profiles indicative of such a response. Thus, the pan-eukaryotic synMuv B genes constitute an element in *C*. *elegans* antiviral defense which is conserved across many eukaryotes where it also may act in viral defense. The enhanced RNAi and conservation of the dREAM complex mutants suggests new therapeutic avenues to boost antiviral defenses.

## Abstract 2

Small RNA pathways can regulate eukaryotic antiviral defense. Here, the authors show that a member of the C. elegans dREAM complex regulates the antiviral RNAi pathway in response to Orsay virus infection, and if mutated it can generate a state of antiviral readiness even in the absence of virus.

## Introduction

RNA interference (RNAi) is a key eukaryotic defense against viruses. In the nematode *Caenorhabditis elegans*, the RNAi pathway is multifaceted, with an expanded set of 25 Argonaute proteins of many classes compared to the 8 Argonautes from 2 classes of many animals, including humans. Argonaute proteins bind to small RNAs, including siRNAs or miRNAs of 22 to 26 nucleotides (nt) which guide those Argonaute/siRNA complexes to target mRNAs with complementarity to the siRNAs to induce their degradation or other forms of regulation such as regulation of translation by most miRNAs. In plants, fungi, nematodes, bivalves, and some insects, but not in most animal species, there is a second-stage amplification of siRNAs by the RNA-dependent RNA polymerase (RdRP) proteins. RdRPs work in concert with Dicer, which initially generates 22 to 26 nt primary siRNAs with a 5′ OH which are extended by RdRPs to produce a far more abundant pool of 22 nt secondary siRNAs with 5′ phosphates. This amplification process relies on RdRP using mRNA templates to extend primary siRNAs, often targeting transposons, integrated viruses, and newly invading viral elements. Mutations in either Argonaute or RdRP genes can disable this RNAi defense [1]. This two-stage RNAi system of *C*. *elegans* is the probable ancestral state of eukaryotic RNAi, with the as yet unexplained deletion of the RdRP genes from most animals (such as humans) and many fungi, but its retention in all plants and a rather peculiar set of animals that includes the nematodes.

Because of this 2 stage siRNA production pathway, RNAi in *C*. *elegans* is remarkably potent, and can even be triggered simply by ingestion from the intestine of double-stranded RNA (dsRNA) produced in an *Escherichia coli* strain engineered using T7 RNA polymerase promoters flanking a cloning site to produce an approximately 1 kb long dsRNA derived from a *C*. *elegans* genomic or cDNA clone [2]. Extensive genetic screens for RNAi-defective (Rde) *C*. *elegans* mutants revealed loss of function mutations in dozens of genes that disable RNAi, including in the Argonaute protein gene *rde-1* (RNAi defective mutant 1) and a collection of *mutator* genes defined before the discovery of RNAi as mutations that activate transposition of *C*. *elegans* transposons which are retroviral elements, genes with a viral history [3,4]. Given that even ingestion of dsRNA can trigger potent RNAi, it was a surprise that soon after the discovery of RNAi mutations emerged from genetic screens that actually enhance the capacity of *C*. *elegans* to silence mRNAs: mutations in a distinct set of 20 genes emerged from genetic screens for enhanced response to dsRNAs that actually enhance the already potent RNAi of *C*. *elegans* [5–13]. A significant fraction of the genes that enhance RNAi (Eri) are members of the synMuv B class genes that were previously identified in totally unrelated genetic screens in the Horvitz and Han labs for increased EGF/Ras signaling in patterning the *C*. *elegans* cell lineage during development. Among the initial collection of multivulva or Muv mutants from 40 years ago was a set of mutants that carried 2 loss of function mutations, in *lin-8* and *lin-9*, or *lin-15A* and *lin-15B*, and required loss-of-function alleles of both genes in order to show the Muv phenotype of increased EGF/ras signaling in the ventral hypodermal lineages [14–16]. Further genetic screens for new mutations that were synthetic Muv with either the *lin-8* (a synMuv A mutant) or *lin-9* (a synMuv B mutant) mutants, but not as single new mutations, revealed a total of about a dozen synMuv B genes that interact with this growth factor signaling pathway. These genes were classified as either a synMuv A (for example, *lin-8* and *lin-56*) or a synMuv B mutation; only a combination of synMuv A plus a synMuv B mutant caused a multivulval phenotype [14].

Molecular analyses of many synMuv B genes mostly by the Horvitz lab showed that many of them encode homologues of the dREAM complex, which is conserved across animals and plants. These include LIN-35, an orthologue of the human tumor suppressor gene retinoblastoma (Rb), and the Rb complex components LIN-53/RbAp48, LIN-37/MIP40, LIN-54/MIP120, DPL-1/DP, and LIN-9/MIP130 [15–21]. Molecular analyses of synMuv A genes showed that *lin-8*, *lin-56*, and *lin-15A* encode widely expressed nuclear proteins with no obvious homologues outside of nematodes [17,18]; the synMuv A genes either evolve very quickly or have evolved in nematodes or lost in other taxons.

The Muv phenotype of the synMuv A; synMuv B double mutants is caused by abnormally high expression of the LIN-3 growth factor ligand in the hypodermal cells that signal to the adjacent vulval cells. Loss of function mutations in any synMuv B gene in combination with any synMuv A mutant cause excessive LIN-3 EGF growth factor signaling from the hypodermal cells to induce a Muv phenotype in the adjacent vulval cell lineages via the EGF receptor and downstream kinase signaling in those cell lineages [15,18,22,23]. Tissue-specific analysis of synMuv A and synMuv B gene activities strongly implicated their function in the hypodermis to regulate the production of the ligand LIN-3 and for this EGF growth factor signaling pathway in the adjacent vulval cell lineages [24,25]. Tissue-specific expression of the *lin-35* synMuv B gene only in the hyp7 hypodermis is sufficient to rescue the Muv phenotype of a *lin-8; lin-35* double mutant, whereas expression of *lin-35* in the ventral hypodermal cells where the LIN-3 EGF signals are received does not rescue the Muv phenotype [22]. In situ mRNA hybridization of *lin-3* shows a dramatic increase in *lin-3* mRNA in the hypodermis only in a synMuv A; synMuv B double mutant, but not in either single mutant [15].

SynMuv B dREAM complex components constitute about 1/3 of the genes that have emerged from screens for enhanced RNAi [5,9,10,12]. The previously characterized roles of the synMuv B dREAM complex in cell cycle and transcriptional regulation/chromatin does not obviously intersect with production or response to siRNAs produced during RNAi; the molecular basis of how the DNA-binding proteins and associated proteins of the synMuv B pathway actually intersect with the Argonaute proteins and RdRPs that are central to RNAi has not yet emerged. Further, while the analysis of the synMuv A and synMuv B mutants in EGF signaling from the hypodermis to the ventral precursor cells has been extensive and beautiful, how it connects to the Eri response of the synMuv B mutants is not at all obvious from this molecular genetic analysis of LIN-3 signaling. There are some differences between the hypodermal to vulval cell signaling by *lin-3* and the enhanced RNAi of synMuv B mutants: while both a synMuv A and a synMuv B class mutation are needed to increase LIN-3 signaling from hyp7 for vulval induction, only a synMuv B mutation is required to enhance RNAi [7], and there is no entanglement of the synMuv A mutations in either the enhanced or defective RNAi phenotypes.

The pleiotropies of *C*. *elegans* dREAM complex mutations suggest a function in the intestine and other non-germline tissues on the production of P-granules, the phase-separated granules initially studied by Priess [26], and by Brangwynne and Hyman, which mediate RNAi, for example Mutator-granules and Z-granules [27,28]. Loss of function mutations in many different *C*. *elegans* synMuv B genes cause misexpression of these normally germline-specific P-granules in somatic tissues, most dramatically in the polyploid intestine and hypodermis [7,29,30]. Because RNAi is normally far more active in the germline [31], the somatic activation of the germline P-granule production is consistent with the increased response to dsRNAs that target somatic cells in the synMuv B mutants.

The P-granule misexpression in the somatic intestine is also salient to RNAi because many RNAi components are localized to these perinuclear, phase-separated organelles, and many proteins and RNAs that mediate RNAi localize to P-granules and to the adjacent Mutator granules [32] and Z granules [33], which are also central to RNAi. The gene expression changes in synMuv B mutants supports the model that they regulate particular genomic client loci that mediate RNAi. For example, in L1 stage *lin-35(n745)*/retinoblastoma mutant animals with just 2 quiescent germline precursor cells (Z2 and Z3) that divide much later in development to generate thousands of germline cells, the P granule genes *pgl-3* (up 28×) and *pgl-1* (up 10×) and the heritable RNAi Argonaute factor *hrde-1* (8×) are significantly up-regulated compared to wild-type L1 stage [34]. These same dramatic gene inductions were also observed in multiple other synMuv B class mutants [29]. But this is not a misspecification of intestinal cell fate to germline fate: the animal is viable and the function of the intestine for nutrition and for vitellogenin synthesis and export to the oocyte is intact. This synMuv B mutant intestinal and hypodermal misexpression of P-granules does not depend on a second synMuv A mutation, unlike the increased EGF signaling in the vulval precursor cells [15].

A common theme to the tissues most affected by the synMuv B mutations, the hypodermis and the intestine, is dramatic endoreduplication. The adult *C*. *elegans* intestine is normally endoreduplicated from diploid at hatching to 32C, 5-inferred full genome duplications; with one duplication at each larval stage but no chromosome condensation or mitosis [25]. The hyp7 hypoderm where ectopic P granules are also observed is 8C [35]. Thus, 2 endoreduplicated tissues are affected by dREAM complex genes. Local gene amplification by the dREAM complex has also been observed in *Drosophila* where the dREAM complex binds at the *Drosophila* chorion genes to control a 50 kb endoreduplication to nearly 100× the gene dosage [36]. This enables the production of the abundant eggshell proteins; mutations in the dREAM complex cause a humpty dumpty phenotype, fragile eggs due to insufficient production of chorion proteins [37–39].

The enhanced RNAi phenotype of the many synMuv B and other Eri mutants, and the siRNA and gene expression changes associated with *eri*-gene mutations points to an elaborate regulation of RNAi activity; the synMuv B mutations which hyperactivate RNAi may place this regulated pathway in a constitutively ON antiviral state, when it is normally a transient state during an actual viral infection. That RNAi may be highly regulated is not surprising: RNAi has evolved by selection for potent antiviral defense during almost a billion years of eukaryotic infection by viruses. But it is surprising that the dREAM complex is so central to RNAi.

Here, we show that *C*. *elegans* synMuv B mutations activate viral defense pathways, leading to gene expression normally associated with viral infection, but in the absence of virus exposure. We show that Orsay RNA virus infection of *C*. *elegans* causes highly congruent gene expression responses to those of synMuv B mutants. Most importantly, we show that a viral infection induces a physiological state that down-regulates synMuv B gene activity: like the synthetic multivulval phenotype that is normally only observed when *C*. *elegans* carries both a synMuv A and synMuv B mutation, Orsay viral infection of a synMuv A mutant causes a partially penetrant Muv phenotype. Importantly, this physiological state confers resistance to Orsay virus: infected synMuvB mutants support 50 to 100× lower Orsay virus RNA levels compared to wild-type animals. These data together show that the down-regulation of synMuv B gene activity or dREAM function is a key defense response that is triggered during an actual viral infection. Thus, the mammalian-conserved synMuv B pathway highlights new avenues to activate production or response to siRNA and may be targeted with drugs to activate human antiviral responses or responses to RNAi-based pharmaceuticals.

## Results

### synMuv B null mutants turn on many genes that are up-regulated during viral infection

Enhanced RNAi (Eri) mutants were first identified by their activation of RNAi in neurons, for example, which normally do not recapitulate the phenotypes caused by loss of function mutations in the corresponding genes. For example, RNAi of various synaptic components often cause no phenotype in wild type, even though genetic mutations cause strong movement disorders; for still unknown reasons neurons in wild type are deficient in RNAi [40]. The *eri* mutants also show enhanced responses to a set of feeding dsRNAs that were discovered over the years which did not recapitulate expected loss of function phenotypes; *lin-1*, *hmr-1*, *lir-1*, *cel-1*, *unc-73*, or *dpy-13* RNAi do not cause phenotypes in wild type but strong phenotypes in *eri* mutants [10]. One model for the existence of the *eri* mutants is that each of them constitutively activates what is normally a highly transient state of increased RNAi, for example, during a viral infection. We explored whether the Eri response of synMuv B mutants might be evidence of their involvement in the orchestration of a coordinated genetic response towards a perceived viral threat, rather than a mere byproduct of gene expression-related abnormalities.

To explore this hypothesis, we analyzed mRNA seq data sets for *C*. *elegans* synMuv B *lin-35(n745)* and *lin-15B(n744)* null mutants (available through the NCBI GEO database collection), for gene expression signatures that may show similarities to the gene expression responses to infection of *C*. *elegans* by the Orsay RNA virus. We analyzed the mRNA seq data sets of developmental-stage matched wild-type N2 and synMuv B *lin-35* and *lin-15B* mutants for the induction of genes that are strongly induced during Orsay viral infection (GEO accession numbers in Materials and methods). About 90 *C*. *elegans* genes are 3- to 10-fold up-regulated when animals are infected by the Orsay RNA virus compared to control animals [41]. A highly overlapping set of 50 genes are 4- to 10-fold up-regulated in *lin-15B* or *lin-35* L1 stage animals compared to wild-type L1 animals. For example, 20 of the 50 *lin-15B* mutant-induced genes and 22 of the 50 *lin-35* mutant-induced genes are also induced in wild-type animals upon Orsay virus infection. Many of the most highly virus-induced *C*. *elegans* response genes and most highly induced *lin-15B* or *lin-35* mutant response genes are the *pals* genes [42,43], which are also dramatically up-regulated in both *lin-35(n745)* and *lin-15B(n744)* null mutants (Table 1 and S1 Fig). Each of the differentially expressed genes shown in Table 1 and S1 Fig are 5-fold or more induced in the synMuv B mutants compared to wild-type controls in the absence of any viral infection. We list the top 50 genes that are up by at least 5-fold in *lin-15B(n744)* and *lin-35(n745)*, mutants in S1–S3 Tables. Perhaps indicative of a local chromatin regulatory mechanism, the 39 PALS genes are localized in five, 20 to 50 kb clusters in the *C*. *elegans* genome [42–45]. Significantly, a mutation in the *pals-22* gene causes increased transgene silencing, a common enhanced RNAi mutant phenotype, including the synMuv B mutants [42–45]. The clustering of these genes may be related to their potential co-regulation or co-amplification during a response to a viral infection.

**Table 1:** Differentially expressed *C*. *elegans* genes upon Orsay virus infection are up-regulated in *lin-35(n745)* and *lin-15B(n744)* synMuv B mutants under non-infection conditions. Listed here are 17 genes previously identified as top statistically significant but functionally uncharacterized in Orsay-infected *C. elegans* [43], and their level of expression within and their level of expression within *lin-35(n745)* or *lin-15b(n744)* synMuv B mutants under non-infection conditions within the mRNA seq data sets *GSM4697089- lin-35[JA1507(n745) rep1/L1*, *GSM4697090- lin-35[JA1507(n745) rep1/L1]*, *GSM1534086- lin-35[JA1507(n745) rep1 L3 and GSM1534087- lin-35[JA1507(n745) rep1 L3]*, and *GSM4697102- lin-15b(n744)- rep1/L1*, *GSM4697105- lin-15b(n744)- rep2/L1*], that were obtained from the NCBI Geo collection. The log2 gene expression Fold changes shown for N2 animals upon Orsay virus infection were obtained from Chen and colleagues [40].

Gene Name	log2 FC in *lin-35(n745)*, L1 Stage	FDR	log2 FC in *lin-35(n745)*, L3 stage	FDR	log2 FC in *lin-15b(n744)*, L1 stage	FDR	Log2 FC in WT(N2) upon Orsay virus infection	FDR
*B0507*.*10*	4,52	1,51E-05	5,87	6,90E-04	4,46	1,07E-04	5,8	1,41E-17
*B0507*.*8*	4,93	3,69E-05	6,57	1,15E-03	4,22	8,69E-04	6,45	4,01E-31
*pals-6*	3,24	1,37E-04	5,41	6,75E-04	3,04	1,78E-03	4,09	9,08E-50
*F26F2*.*4*	3,08	1,17E-02	9,47	5,38E-03	N/A	N/A	7,79	4,09E-116
*sdz-6*	6,12	5,14E-03	8,72	1,27E-02	5,26	5,82E-02	5,63	2,52E-47
*F26F2*.*2*	N/A	N/A	N/A	N/A	N/A	N/A	N/A	N/A
*F26F2*.*3*	N/A	N/A	7,13	2,69E-05	N/A	N/A	8,37	6,06E-127
*C43D7*.*4*	4,58	4,57E-03	7,96	1,89E-02	3,42	1,20E-01	9,99	1,22E-61
*pals-28*	3,98	4,44E-03	6,6	1,84E-02	4,99	4,52E-03	8,31	8,32E-55
*F26F2*.*5*	N/A	N/A	8,59	2,10E-03	N/A	N/A	7,45	1,64E-58
*F42C5*.*3*	4,8	1,33E-05	8,59	2,10E-03	4,37	2,00E-04	6,49	9,12E-90
*pals-5*	7,59	8,19E-07	7,89	3,47E-04	9,06	5,80E-07	6,72	2,47E-65
*F26F2*.*1*	N/A	N/A	4,87	5,55E-04	N/A	N/A	5,76	2,52E-62
*eol-1*	4,48	8,42E-07	4,67	2,45E-04	3,89	1,80E-05	6,06	2,32E-79
*Y57B8A*.*39*	N/A	N/A	N/A	N/A	N/A	N/A	N/A	N/A
*C49C8*.*2*	N/A	N/A	N/A	N/A	N/A	N/A	N/A	N/A
*C43D7*.*7*	N/A	N/A	N/A	N/A	N/A	N/A	8,85	1,39E-32

**Fig 1:**
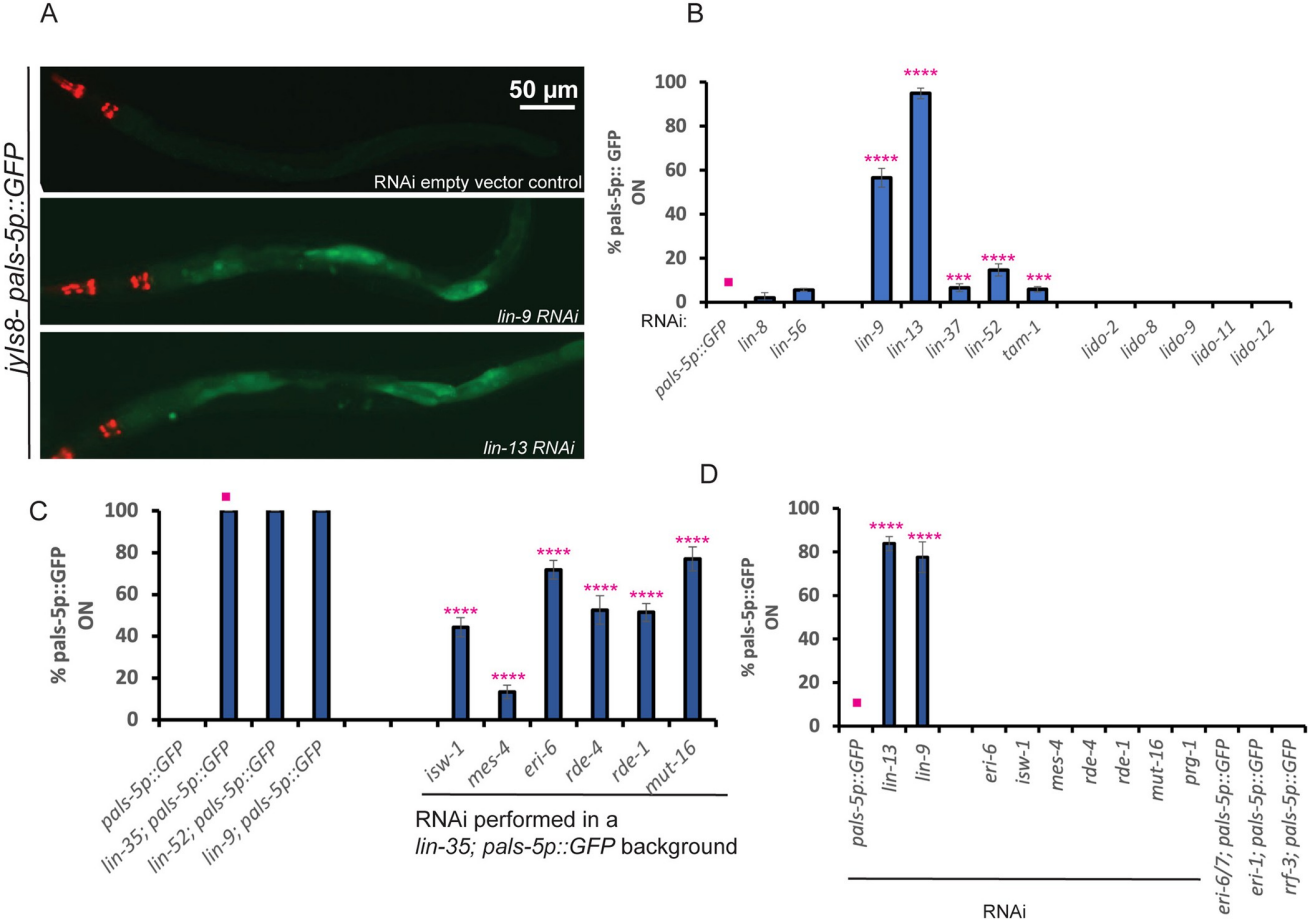
A *pals-5p*::*GFP* fusion gene is induced in synMuv B mutants in the absence of viral infection. (A) Fluorescent micrographs showing *pals-5p*::*GFP* expression in animals raised on *E*. *coli* expressing dsRNA of empty vector control L4440 or dsRNA targeting *lin-9* or *lin-13* synMuv B genes. Scale bar is indicated. (B) Quantification of the percentage of animals showing *pals-5p*::*GFP* expression when raised on *E*. *coli* expressing dsRNA against a panel of different synMuv B *eri-*genes including *lin-9*, *lin-13*, and non-Eri synMuv A genes *lin-8*, *lin-56*, and a panel of *lido (for* **li**n-8 **do**main) genes (C). Quantification of the percentage of animals showing *pals-5p*::*GFP* expression in *lin-35(n745); pals-5p*::*GFP*, *lin-9(n112); pals-5p*::*GFP* and *lin-52(n771); pals-5p*::*GFP* synMuv B mutants and percentage of *lin-35(n745); pals-5p*::*GFP* animals showing *pals-5p*::*GFP* expression when raised on *E*. *coli* expressing dsRNA against the synMuv Eri suppressor genes *isw-1* and *mes-4* and other key genes that encode components central to RNAi such as *rde-1*, *rde-4*, and *mut-16* (D). Percentage of animals showing *pals-5p*::*GFP* expression when raised on *E*. *coli* expressing dsRNA against the underlined panel of genes in the *jyIs8[pals-5p*::*GFP] animals*. Non-underlined genes are genetic doubles that were generated in the *pals-5p*::*GFP* background with strong loss-of-function mutations of the genes shown. Statistical comparisons were made between samples marked with asterisks and the sample denoted by a solid pink dot. **** indicates *p* < 0.0001, and *** indicates *p* < 0.001, as determined using one-way ANOVA followed by Dunnett’s post hoc test. The numerical data underlying the graphs shown in this figure can be found in the Supporting information data table named S1 Raw Data. Raw microscopy images corresponding to panel (A) have been deposited in Zenodo and are accessible at DOI: 10.5281/zenodo.14289232.

A *pals-5* promoter fusion to GFP has been developed as a reporter of viral infection response by the Wang and Troemel labs after their RNA seq of viral and microsporidia infection showed that *pals-* genes are highly induced [42]. The strong induction of *pals-* genes in the *lin-15B* or *lin-35* synMuv B mutants may report the aberrant induction of antiviral defense in the absence of a real viral infection in these mutants. For example, a *pals-5p*::*GFP* fusion gene is not expressed in wild-type *C*. *elegans* not infected with Orsay virus, but is strongly activated in the intestine of a wild-type animal carrying the *pals-5p*::*GFP* after Orsay virus ingestion, the mode of *C*. *elegans* Orsay viral infection [42]. To explore this viral response in the synMuv B mutants, we used RNAi or mutations to disrupt particular synMuv B genes in a strain carrying *pals-5p*::*GFP*. RNAi of the synMuv B genes *lin-9* or *lin-13* strongly activated *pals-5p*::*GFP* (Fig 1A and 1B). We also crossed strong loss-of-function mutations of *lin-35(n745)*, *lin-52(n771)*, and *lin-9(n112*), into a strain carrying *pals-5p*::*GFP* and found that all the 3 synMuv B homozygous mutants robustly activate *pals-5p*::*GFP* (Fig 1C). We further tested by RNAi the synMuv A genes *lin-8* or *lin-56* as well as several *lido* (LIN-8 domain containing) genes, paralogues of the synMuv A gene *lin-8* [46], but these gene inactivations of synMuv A genes or their paralogues did not induce *pals-5p::GFP* (Fig 1B). These findings support the hypothesis that the *C*. *elegans* synMuv B mutants may place the animal in a physiological state that is normally induced by a viral infection, triggering the activation of their antiviral response, for example, induction of specific viral defense genes and activation of RNAi.

Also suggesting that the *pals* gene family act in a pathway of anti-viral defense, multiple *pals-22* null mutations were identified in a genetic screen for silencing of multicopy transgenes, which are recognized by the RNAi pathway as foreign genetic elements akin to viruses and silenced by the pathway [45]. Transgenes are recognized as foreign, most especially in Eri mutants such as the synMuv B and other Eri mutants (a concept that is further assessed in later experiments discussed in this study) [10]. The *pals-22* allele that emerged from a screen for transgene silencing also showed enhanced RNAi with the *dpy-13* and *cel-1* feeding RNAi that are standard tests for enhanced RNAi [45]; thus, the *pals-22* loss of function mutant is an Eri mutant. The induction of the *pals* genes by viral infection and by the synMuv B mutations in *lin-15B* or *lin-35*, and the observed enhanced RNAi of *lin-15B* or *lin-35* null mutants is consistent with association between *pals-* genes and antiviral defense.

### Gene inactivations that suppress the Muv phenotype of synMuv B; synMuv A double mutants also suppress *pals-5p*::*GFP* induction by synMuv B loss of function mutations

The Han group discovered 32 gene inactivations that can suppress the Multivulvae (Muv) phenotype of synMuv A; synMuv B double mutants [14]. Seven of those Muv suppressor gene inactivations, *mes-4*, *isw-1*, *gfl-1*, *mrg-1*, *ZK1127.3*, *M03C11.3*, and *C3410.8* were nearly 100% suppressors of the Muv phenotype by RNAi. A subset of these synMuv suppressors, also suppress the Eri phenotype of synMuv B mutants [14]. Two of the more potent synMuv B Eri suppressors include *isw-1*, which encodes a member of the ISW1 complex and *mes-4*, which encodes a Set domain containing protein, fitting with the chromatin annotation of the dREAM complex [14]. We tested whether the induction of *pals-5p*::*GFP* in synMuv B mutants is functionally linked to their Eri phenotype. We reasoned that suppressing the Muv or the Eri phenotype of synMuv B mutants could also cause a decrease in the induction of the *pals-5p*::*GFP* reporter. To test this, we targeted *isw-1* and *mes-4* Muv suppressors by RNAi in the *lin-35(n745); pals-5p*::*GFP* strain. As controls, we also targeted other core components of the RNAi machinery such as the Argonaute gene *rde-1*, the RNA helicase gene *rde-4* and the mutator protein gene *mut-16* and determined the percentage of GFP-positive animals in their F1 progeny. We found that knocking down *isw-1*, *mes-4*, *rde-1*, *rde-4*, and *mut-16* by RNAi partially but not completely suppressed the percentage of GFP-positive animals in the *lin-35(n745); pals-5p*::*GFP* animals (Fig 1C). We also showed that inactivation of *isw-1*, *mes-4*, *rde-1*, *rde-4*, or *mut-16* by RNAi in an otherwise wild-type background does not activate *pals-5p*::*GFP* (Fig 1D).

We also characterized the induction of *pals-5p*::*GFP* in enhanced RNAi mutants that are not synMuv B genes and found that only synMuv B mutations or synMuv B gene RNAi induce *pals-5p*::*GFP*. For example, inactivation by RNAi or by mutations of the *eri* genes *eri-6*, *eri-6/7*, *eri-1*, and *rrf-3* does not activate *pals-5*::*GFP* (see Fig 1C and 1D). This suggests that the Eri phenotype induced by synMuv B gene mutations may enhance RNAi via a pathway that is distinct from the other known Eri mutants. In addition, targeting *prg-1*, the worm ortholog of PIWI, which silences integrated viruses in *Drosophila* [47], by RNAi, also did not activate *pals-5*::*GFP* expression (see Fig 1D), suggesting that piRNA-mediated defense response is distinct from that of the Eri phenotype caused by synMuv B mutants.

**Fig 2:**
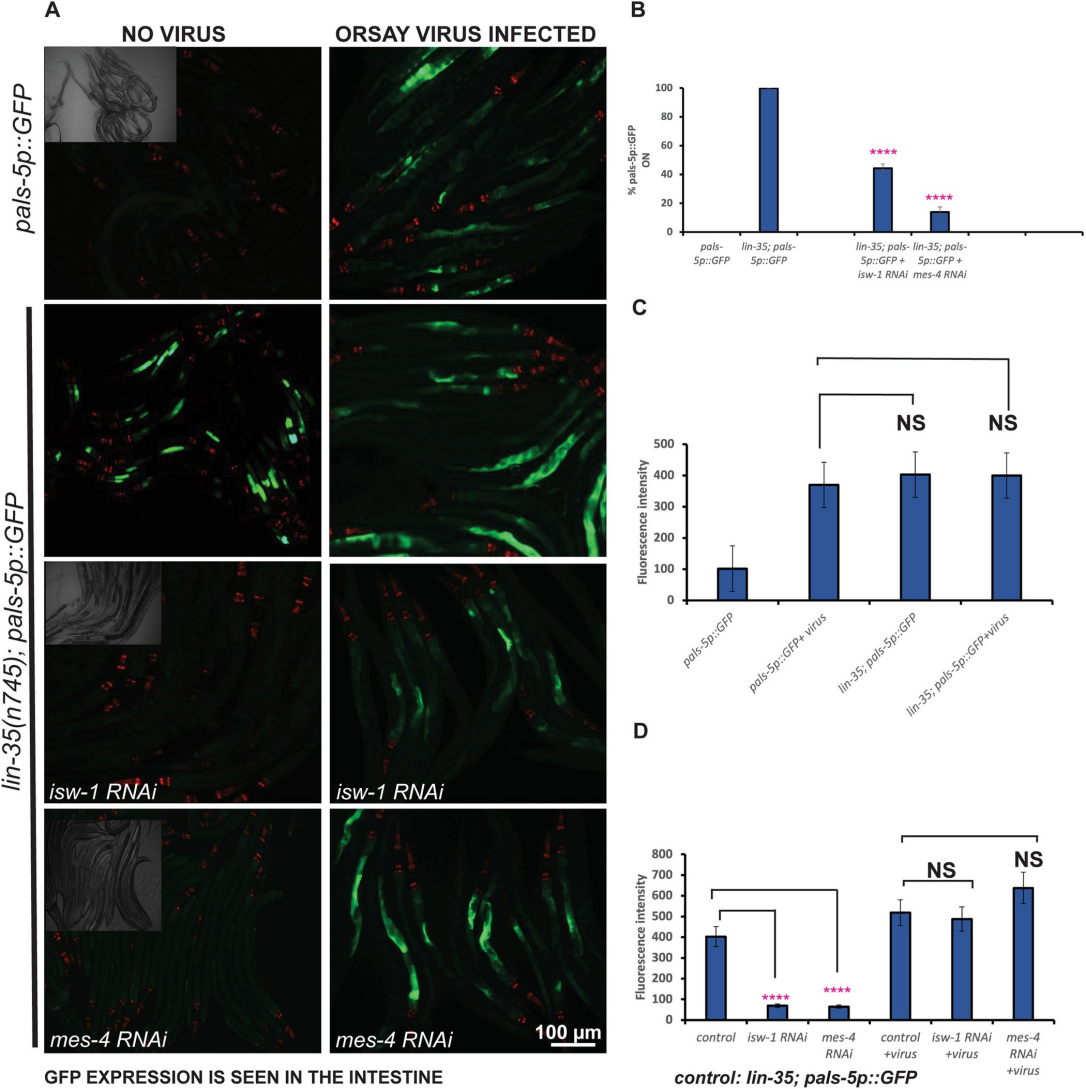
Gene inactivation by RNAi of the synMuv suppressor genes encoding the chromatin remodeling and histone methylation factors *isw-1* and *mes-4* significantly suppresses the activation of *pals-5p*::*GFP* in the *lin-35(n745)* mutant under non-infection conditions but does not suppress *pals-5p*::*GFP* response to Orsay virus infection. (A) Fluorescent micrographs of *pals-5p*::*GFP* (control) or *lin-35(n745); pals-5p*::*GFP* animals not exposed to Orsay virus or after Orsay virus infection. A brightfield image highlights the number of animals that were imaged. Scale bar is indicated. (B–D) Graphs showing a quantification of *pals-5p*::*GFP*(control) or *lin-35(n745); pals-5p*::*GFP* under RNAi of *isw-1* or *mes-4*, as well as failure of isw-1 or mes-4 RNAi to suppress induction of *pals-5p*::*GFP* by Orsay virus infection. **** indicates *p* < 0.0001, as determined using one-way ANOVA followed by Dunnett’s post hoc test. NS: not statistically significant. The numerical data underlying the graphs shown in this figure can be found in the Supporting information data table named S1 Raw Data. Raw microscopy images shown in this figure have been deposited in Zenodo and are accessible at DOI: 10.5281/zenodo.14289232.

We also infected *pals-5p*::*GFP* wild type and *lin-35(n745); pals-5p*::*GFP* animals with the Orsay virus and examined their F2 progeny for *pals-5p*::*GFP* activation. We examined F2 progeny to allow sufficient time for the viral infection to become well-established within the population. *pals-5p*::*GFP* animals infected with the Orsay virus showed a robust activation of *pals-5p*::*GFP*, whereas *pals-5p*::*GFP* animals not infected with Orsay virus did not induce GFP expression (Fig 2A and 2C). The *lin-35(n745); pals-5p*::*GFP* animals show 100% penetrant GFP activation under both infected and uninfected conditions (Fig 2A–2C). Inactivation of *isw-1* and *mes-4* by RNAi in *lin-35(n745); pals-5p*::*GFP* animals under non-Orsay infection conditions significantly suppressed the *pals-5p*::*GFP* reporter activation (Fig 2A, 2B, and 2D). However, upon infection by the Orsay virus, we no longer observed this suppression, which suggests that *isw-1* and *mes-4* function while required for the induction of *pals-5p*::*GFP* in *lin-35(n745)* null mutants under non-infection conditions may be dispensable or insufficient in the context of an actual viral infection.

### *lin-15B* null mutants exhibit altered intestinal nuclear morphology

**Fig 3:**
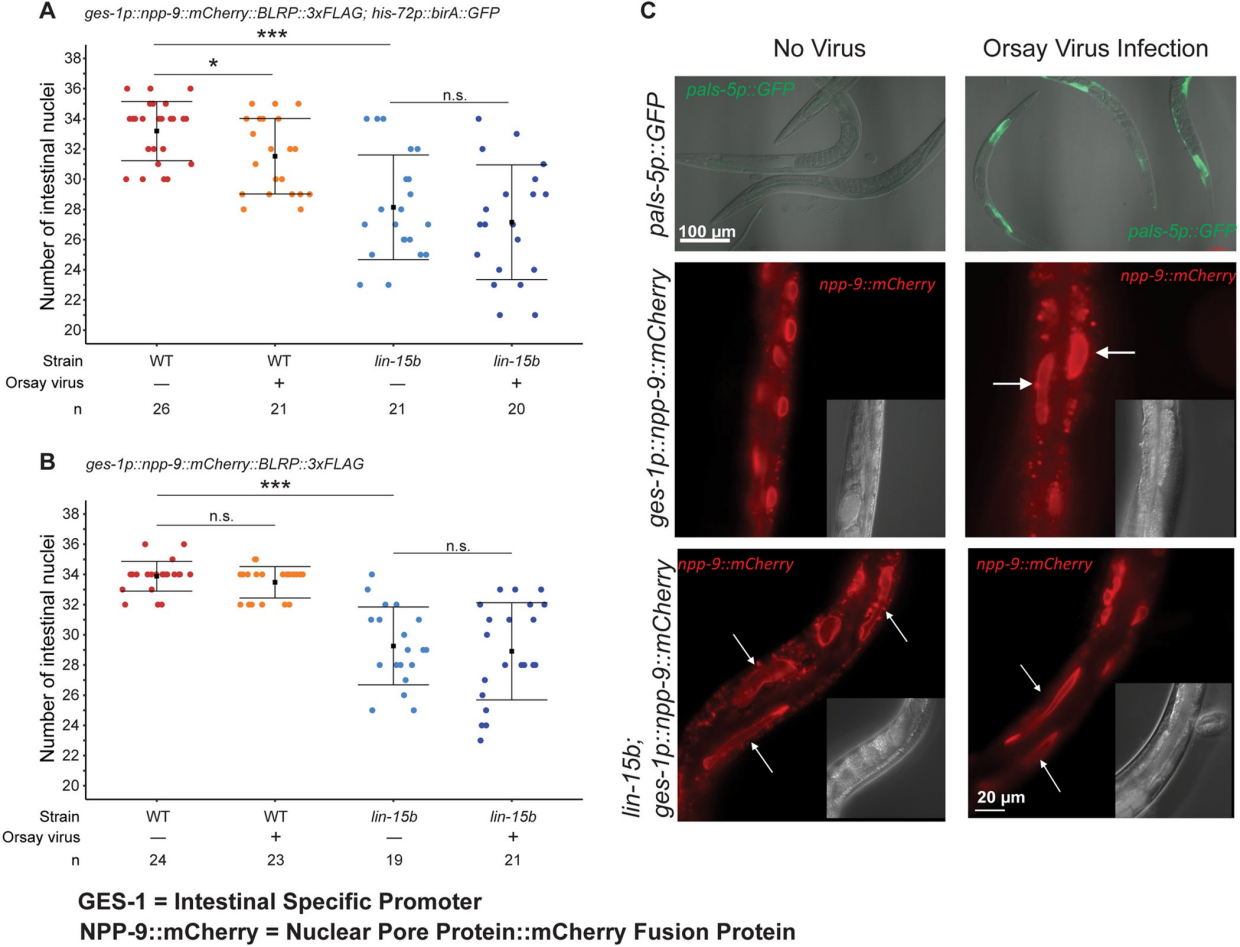
*lin-15b(-)* animals exhibit altered intestinal nuclear morphology. (A) Number of mCherry expressing intestinal nuclei in *FAS207* adults in wild-type and lin-15b(W485*) mutant backgrounds. (B) Number of mCherry expressing intestinal nuclei in *JJ2284* adults in WT and lin-15b(W485*) backgrounds. The square represents the average, and the bars represent the standard deviation. The sample size *n* has been indicated; *p* value calculated using ANOVA with Bonferroni correction. n.s: not statistically significant, * *p* < 0.05, *** *p* < 0.001. (**C**) Representative images of *pals-5p::GFP* (top row), *FAS207*, and lin-15b(W485*); *FAS207* adults. White arrows indicate elongated intestinal nuclei. Scale bar is indicated. The graphs shown in A and B were generated using custom scripts in R studio and the statistical analyses were done using Jupyter Notebook. The numerical data underlying the graphs shown in this figure can be found in the Supporting information data table named S1 Raw Data. The raw microscopy images shown in this figure have been deposited in Zenodo and are accessible at DOI: 10.5281/zenodo.14289232.

*C*. *elegans* infection with Orsay virus causes morphological changes in intestinal cells, including cell fusion, elongation of nuclei, and nuclear degeneration [48]. We looked for similar phenotypes using 2 strains that carry transgenes to visualize the intestinal nuclear membrane using the mCherry tagged nuclear pore protein NPP-9 (*ges-1p*::*npp-9*::*mCherry*::*BLRP*::*3xflag* or *ges-1p*::*npp-9*::*mCherry*::*BLRP*::*3xFLAG; his72p*::*birA*::*GFP*). One set of animals were infected with Orsay virus while the control group received no virus, and both groups were observed for 2 generations. The intensity of viral infection was established by the robust expression of *pals-5p*::*GFP* in animals carrying that transgene and infected simultaneously (Fig 3C, top panel). The no virus infection control animals had an average of 33.2 ± 2 intestinal nuclei at the adult stage. Orsay virus infected adults exhibited a 5% decrease in the number of intestinal nuclei, (31.5 ± 2.5) (Fig 3A and 3B), weakly supportive of the previously observed nuclear degeneration. We also observed elongation of intestinal nuclei in some Orsay virus-infected animals (Fig 3C, middle panels).

To further test the intestinal nuclear response in synMuv B mutants to Orsay virus infection, we constructed a lin-15B(W485*) null mutant allele based on the canonical *lin-15B(n744)* null allele using CRISPR in the strain harboring the transgene *ges-1p*::*npp-9*::*mCherry*::*BLRP::3XFLAG*. We observed that under no virus infection conditions, lin-15B(W485*) animals exhibited a 15% decrease in the number of intestinal nuclei, with an average of 28.1 ± 3.5 nuclei (Fig 3A). L1 larval stage wild-type animals normally have 20 intestinal nuclei which increase to 36 nuclei by the L2 stage, as most L1 intestinal cells undergo mitosis or at least nuclear division. Those 36 nuclei then increase their DNA content by undergoing DNA replication without cytokinesis during the next 2 larval stages and into adulthood, ending up with 36 nuclei with 32C genome copy number as roughly assayed by DAPI staining of DNA [35]. Thus, the minimum number of nuclei we could expect to observe if there was no cell division at the first larval stage is 20; so, a decrease from 33 nuclei in wild type and to 28 nuclei in a *lin-15B* null mutant was substantial.

Strikingly, many of the intestinal nuclei in the lin-15B(W485*) animals were elongated (Fig 3C, lower panels), suggesting this Orsay virus-associated cellular alteration is constitutively present in these *lin-15B(-)* animals. The decrease in the number of intestinal cells and the altered morphology of intestinal nuclei was also observed in a second independent line of lin-15B(W485*) that was generated in the *JJ2284* strain (S8 Fig). Orsay virus infection of the lin-15B(W485*) animals did not cause a further reduction of the number of intestinal nuclei or any change in the intestinal nuclear morphology. This implies that the intestinal nuclear response to viral infection of wild-type *C*. *elegans* may be a direct result of down-regulation of *lin-15B* or other synMuv B genes in Orsay virus-infected animals. Fusion of intestinal cells in Orsay virus-infected animals has been reported previously [48]. Both the observed decrease in the number of nuclei as well as the elongated appearance of the intestinal nuclei could be the result of a fusion of intestinal nuclei. However, we cannot rule out the alternate possibility that the decrease in the number of intestinal nuclei in *lin-15B(-)* animals and Orsay virus-infected animals could be due to a lineage defect or defect in karyokinesis during development.

### Orsay virus infection down-regulates synMuv B gene activity

**Fig 4:**
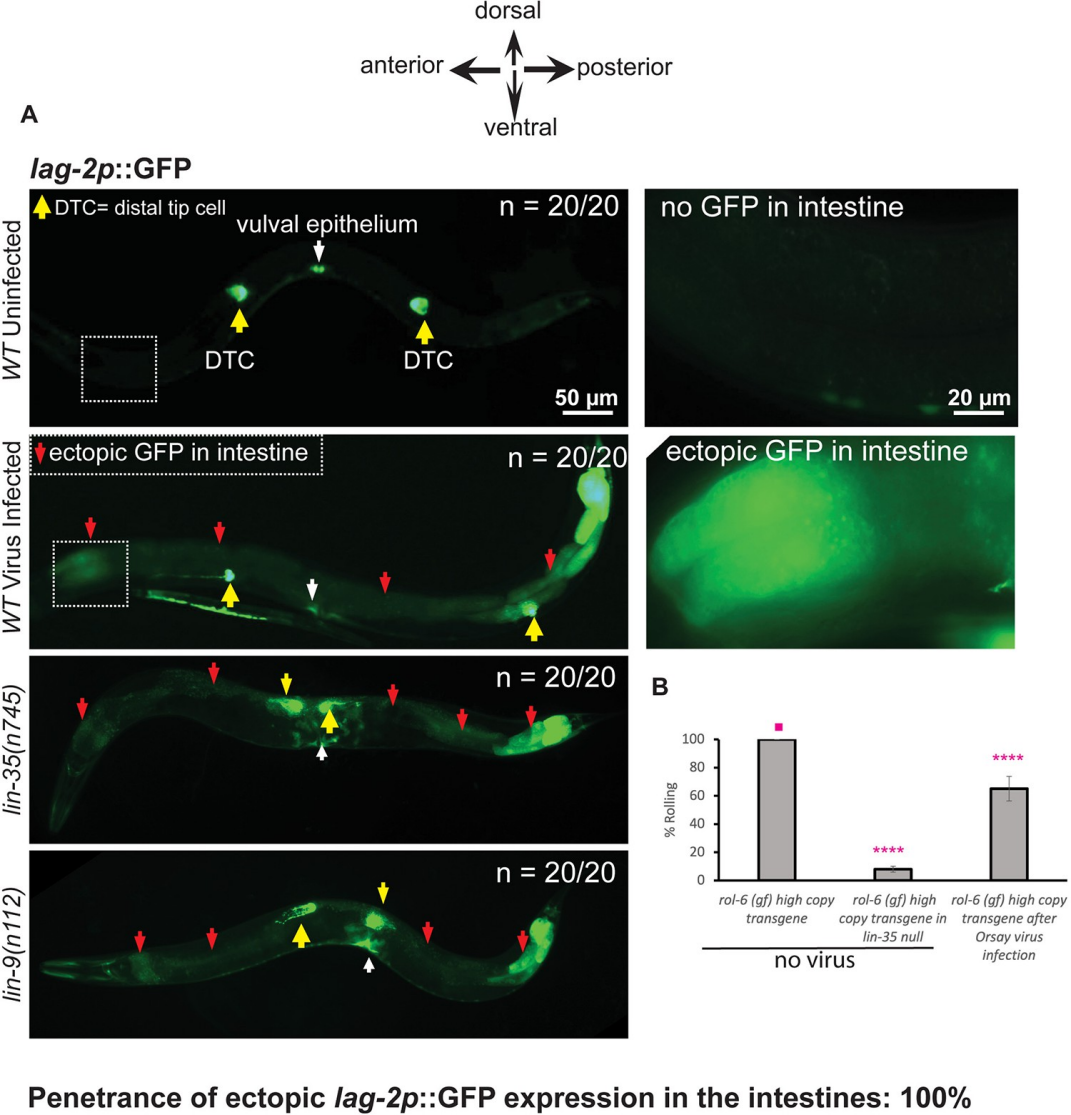
Orsay virus infection causes misexpression of *lag-2p*::*GFP* in somatic tissues. (A) Shown are fluorescent micrographs depicting *lag-2p*::*GFP* expression in worms under no virus and under Orsay virus infection. The position of the distal tip cells of the germline are indicated by yellow arrows and the panels to the right of the WT/uninfected and WT/infected worms are panels of the region that is boxed on the left at a higher magnification. New ectopic expression of GFP seen within intestinal cells in the virus-infected animals is labeled with red arrows. Ectopic expression of GFP within intestinal cells of *lin-35(n745)* and *lin-9(n112)* mutants is shown as additional positive controls and the ectopic expression of GFP in the intestine is labeled with red arrows. The position of the central vulva is labeled with a white arrow. Scale bar is indicated. (B) Shown here is a quantification of the percentage of Rol phenotype of *mgIs30* animals that bear an integrated *rol-6* transgene, under no virus and infection with the Orsay virus. The suppression of the *rol-6* transgene in a *lin-35(n745)* null mutant is shown to provide a quantitative estimate of the intensity of the enhanced RNAi phenotype that is typical of most Eri mutants. Statistical comparisons were made between samples marked with asterisks and the sample denoted by a solid pink dot. **** indicates *p* < 0.0001, as determined using one-way ANOVA followed by Dunnett’s post hoc test. The raw microscopy images shown in this figure have been deposited in Zenodo and are accessible at DOI: 10.5281/zenodo.14289232.

The *C*. *elegans* Delta-like ligand for the GLP-1 and LIN-12 Notch receptors is encoded by *lag-2*. *lag-2* is normally expressed only in the distal tip cells of the gonad and a subset of the vulval precursor cells [49]. But mutations in several synMuv B genes cause nearly 100% penetrant ectopic *lag-2*::*GFP* expression in intestinal cells, which can be suppressed by gene inactivation of synMuv suppressor genes such as *isw-1* [14]. If a *C*. *elegans* viral infection normally triggers the down-regulation of synMuv B genes, perhaps as a natural defense mechanism, we hypothesized that this may cause misexpression of *lag-2* within the intestinal cells as is observed in non-virally infected synMuv B mutants [14]. We infected wild-type animals carrying the *lag-2p*::*GFP* fusion gene with the Orsay virus and scored their F2 progeny for *lag-2p*::*GFP* misexpression in the intestine during the L4/adult stages. Upon infection of wild-type animals with the Orsay virus, *lag-2p*::*GFP* was misexpressed in the intestinal cells with a 100% penetrance, similar to synMuv B mutants that are not infected with Orsay virus (Fig 4A). This virus-induced intestinal misexpression of *lag-2p*::*GFP* was not suppressed by inactivation of synMuv suppressor genes such as *isw-1*, in contrast to the *lag-2p*::*GFP* induction in the synMuv B mutants (S2A and S2B Fig). These data show that infection with Orsay virus recapitulates certain synMuv B loss of function phenotypes, for example, the induction of *lag-2p*::*GFP* in the intestine.

An integrated multicopy transgene (*mgIs30*) carrying a tandemly duplicated collagen mutant allele *rol-6(su1006)*, an R71C substitution mutation of *rol-6* that causes the Rolling phenotype due to collagen assembly defects [50], causes a nearly 100% rolling (Rol) phenotype in wild type. But in nearly all Eri mutants (*eri-1*, *rrf-3*, *eri-6/7*, *lin-35*, *lin-15B*, and other synMuv B mutants), this transgene, like most multicopy transgenes, is recognized as foreign and silenced so that the animals no longer roll [11]. We reasoned that if down-regulating synMuv B genes represent a natural part of the antiviral defense, then a viral infection itself may induce an Eri response, which may silence multicopy transgenes such as the *rol-6* transgene. We infected a wild-type strain carrying the *mgIs30* multicopy *rol-6* transgene with the Orsay virus and scored the percentage of rolling animals in the F2 generation. Consistent with this hypothesis, Orsay viral infection partially silenced the Rol phenotype of *mgIs30* (Fig 4B), though to a weaker extent than what is observed in a synMuv B Eri mutant such as a *lin-35* null mutant, for example, where the silencing of the *rol-6* transgene is nearly 100% (Fig 4B). This difference in the level of transgene silencing may be due to the extent of inhibition of synMuv B gene activity, for example, due to variations in viral titer or the synMuv B pathway response to a viral infection.

### Orsay virus infection of a synMuv A null mutant induces a Muv phenotype

Given that *lag-2p*::*GFP* is strongly misexpressed with a 100% penetrance within intestinal cells in wild-type *C*. *elegans* infected with Orsay virus (which is strikingly similar to the *lag-2p*::*GFP* intestinal expression observed in synMuv B mutants), and the observation that Orsay virus infection in wild-type animals enhances RNA interference, we hypothesized that down-regulation of some or all synMuv B genes may represent a normal step in viral defense. The simplest prediction of this hypothesis is that infecting a synMuv A mutant with the Orsay virus might down-regulate synMuv B activity as part of the normal response to a viral infection, so that, like a synMuv A; synMuv B double mutant, viral infection of a synMuv A mutant may cause a Muv phenotype. Only when both synMuv A and synMuv B genes are mutant does the multiple vulvae or Muv phenotype occur in non-virally infected animals. If a viral infection in animals lowers synMuv B gene activity as a defense mechanism, we predicted that Orsay virus infection of a synMuv A null mutant, just the single synMuv A mutation, might be sufficient to cause a Muv phenotype in some fraction of the population.

**Fig 5:**
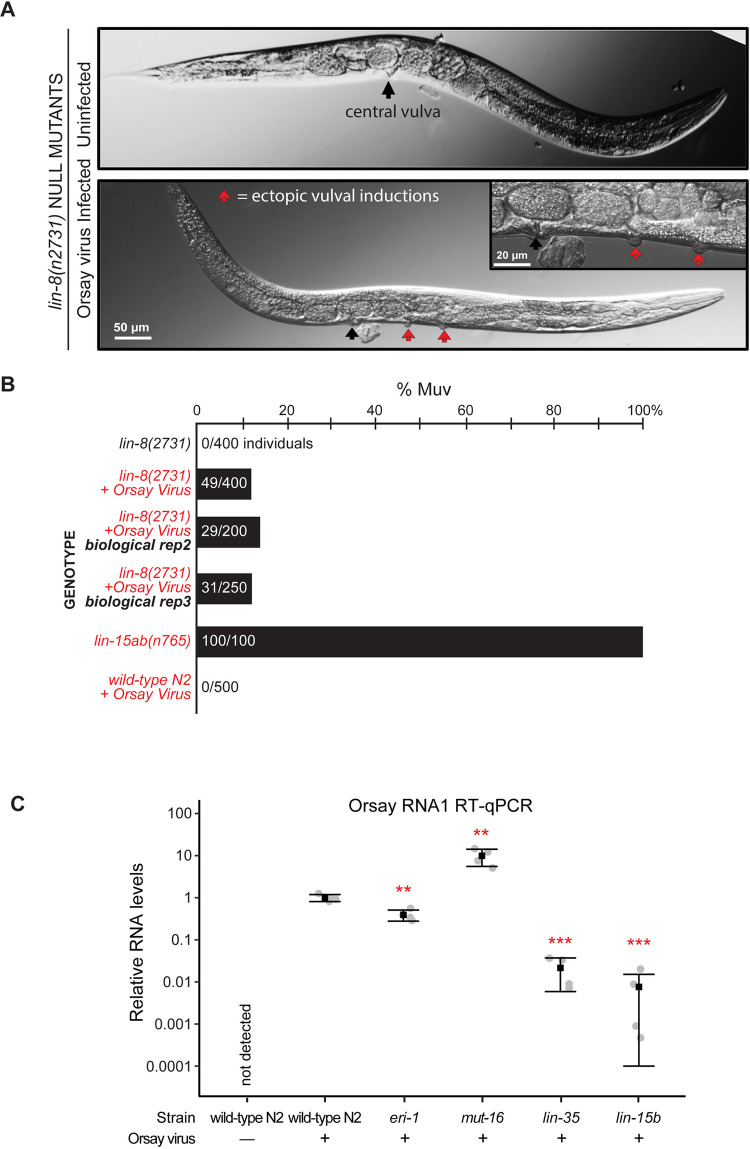
Orsay virus infection of the *lin-8(n2731*) synMuv A mutant induces a Muv phenotype indicative of decreased synMuv B gene activity, and 2 different synMuv B mutants produce 50–100× less Orsay virus RNA. (A) Brightfield micrographs showing uninfected vs. virus-infected *lin-8(n2731)* mutant animals. The central vulva that is normally induced in wild type is indicated by a black arrow. The inset image in the panel showing the virus-infected animal is a magnified region showing ectopic vulval inductions. The ectopic vulval inductions are indicated by red arrows. (B) Quantification of the percentage of Muv animals seen in *lin-8(n2731*) mutants under uninfected compared to Orsay virus infection. The Muv quantification was performed on the F2 generation of the virus-infected animals. Scale bar is indicated. (C) RT-qPCR for Orsay RNA1 in uninfected wild type N2, infected wild type N2, infected *eri-1(mg366)*, infected *mut-16(pk710)*, infected *lin-35(n745)*, and infected *lin-15b(n744)* animals. The infected *lin-35(n745)* and *lin-15b(n744)* animals produce 50× and 100× less Orsay viral RNA, respectively, after Orsay infection. The gray circles indicate 2 technical replicates from 2 biological replicates. The black square represents the average of the 4 values and the error bars represent the standard deviation. Two-tailed unpaired Student’s *t* test *p*-values **p < 0.01, ***p < 0.001. The raw microscopy images shown in this figure have been deposited in Zenodo and are accessible at DOI: 10.5281/zenodo.14289232.

To test this, we infected the synMuv A *lin-8(n2731)* null mutant with the Orsay virus and examined F2 progeny for the appearance of the Muv phenotype. Strikingly, in 3 independent trials, we found that 10% of the F2 progeny were indeed Muv (Fig 5A and 5B), whereas uninfected *lin-8* mutant control animals did not produce any Muv progeny. The appearance of a Muv phenotype has never been reported in wild-type *C*. *elegans* under Orsay virus infection conditions in any previous study nor in our own observations (Fig 5B).

To rule out Orsay induction of a synMuv B mutation, we followed the progeny of these Orsay-infected synMuv A mutant animals. We picked single progeny from 8 gravid *lin-8(n2731)* adult animals that were Muv after an Orsay virus infection, bleach treated them to remove any residual viral particles that may be present, and then allowed their eggs to hatch and examined their progeny at the adult stage to discern if the Muv phenotype persisted. We found that 100% of their progeny had a non-Muv vulval phenotype. Thus, the Orsay virus infection did not induce a mutation in a synMuv B gene in *lin-8(n2731)* null animals to give rise to a Muv phenotype.

Taken together, these findings suggest that down-regulation of one or more synMuv B genes is a normal step in the antiviral defense response, and that viral infection induces an enhanced RNAi physiological state. The 10% penetrance of the Muv phenotype suggests that the down-regulation of the synMuv B gene activity is just at the threshold for synergizing with the synMuv A mutations to confer a Muv phenotype (see Discussion).

### Down-regulation of synMuv B gene activity increases viral resistance

In response to Orsay virus infection, synMuv B gene activity is down-regulated, raising the question of whether this reflects an adaptive antiviral mechanism or a pathogenic consequence of infection. To investigate this further, we infected wild-type or the synmuv B mutants *lin-35(n745)* or *lin-15b(n744)* with Orsay virus filtrate at the L1 stage and measured levels of Orsay RNA1 in adults by RT-qPCR, as a proxy for infection levels. If synMuv B gene down-regulation is part of the antiviral response, we predicted that *synMuv B* mutant animals would display lower infection levels as measured by viral RNA levels. Conversely, if the down-regulation is a pathogenic consequence, mutant animals would exhibit infection levels equal to or greater than those of wild type. *lin-35* mutant animals had 50-fold lower and *lin-15b* mutant animals had 130-fold lower Orsay RNA1 than wild-type animals (Fig 5C). These results suggest that synMuv B mutants are more primed for viral defense, with the reduced synMuv B activity conferring an enhanced antiviral response, perhaps evidence that the animal is in an antiviral physiological state of low synMuv B gene activity.

Given that small RNA pathways are central to the antiviral response in *C*. *elegans* [48,51,52], we further analyzed Orsay viral RNA levels in *eri-1(mg366)*, an RNAi-enhanced mutant [40], and *mut-16(pk710)* [3,30], an RNAi-deficient strain. As anticipated, *mut-16* mutants displayed a 10-fold increase in Orsay viral RNA levels, underscoring the essential role of small RNAs in viral defense. Interestingly, *eri-1* mutant animals showed only a 2.5-fold reduction in Orsay viral RNA compared to wild type (Fig 5C). This suggests that loss of synMuv *B* activity results in a far more robust antiviral response than the loss of *eri-1*. While synMuv *B* mutants also exhibit enhanced RNAi phenotypes [7], the specific small RNA populations or tissues involved in this stronger antiviral response remain to be determined.

### The siRNA landscape of synMuv B Eri mutants

Abundant endogenous siRNAs derived from thousands of genes are produced by wild-type *C*. *elegans* that are not infected with viruses or exposed to specific dsRNAs that trigger additional exogenous RNAi of one targeted gene. These natural siRNAs regulate the mRNA levels and chromatin structures of a huge number of genes in wild-type *C*. *elegans;* nearly all of these siRNAs are not produced in the RNAi-defective *mut-2* or *mut-16* mutants, and stunningly, these missing siRNAs from thousands of genes cause almost no phenotypes except for activation of normally dormant transposons and a failure to respond to injected or ingested dsRNAs [3,4,53,54]. In contrast, enhanced RNAi mutants disrupt the production of a far smaller subset of endogenous siRNAs. *C*. *elegans* siRNAs are classified by their length and 5′ nucleotide, which generally predict which of the nearly 2 dozen Argonaute proteins present them to specific mRNA and other target RNAs [55]. The primary 26G siRNAs (26 nt in length with G at the 5′ end), associate with the Argonaute proteins ERGO-1 and ALG-3/4. The targets of ERGO-1 26G siRNAs include about 100 protein coding genes, pseudogenes, and long noncoding RNAs, many of which are integrated retrotransposons [54]. The *ergo-1* mutants are strongly enhanced for RNAi, suggesting that either silencing these retrotransposons uses much of the RNAi bandwidth of wild-type *C*. *elegans*, or that the viral activation that results from failure to silence those retrotransposons with ERGO-1 siRNAs when *ergo-1* is defective, in turn enables a viral infection from those normally silenced retroviruses, to in turn induce an antiviral response, or increased RNAi, a sort of *C*. *elegans* inflammatory response. Targets of the ALG-3/4 26G siRNAs include spermatogenic genes [56–59]; these 26G siRNAs and the Argonaute that mediate their recognition of mRNAs in sperm have not been implicated in normal RNAi or enhanced RNAi, and the 100× to 1,000× more abundant 22G siRNAs (22 nt in length with G at the 5′ end), are loaded into the many WAGO Argonautes, which are greatly expanded in the nematodes compared to other animals, and CSR-1. A large fraction of these 22G siRNAs is produced in the germline to mediate the massive level of gene silencing in the germline and to ensure that silenced genes from the parent continue to be silenced in progeny. CSR-1-associated 22G siRNAs are also expressed in the germline and modulate expression of germline genes as well as genes that mediate chromosome segregation at meiosis and mitosis [60,61]. The WAGO-associated 22G siRNAs target protein coding genes, pseudogenes, and transposons [27]. Almost all 22G siRNAs are depleted in the *mut-16* RNAi defective mutant, but most of these very abundant siRNAs are produced normally in the enhanced RNAi mutants *eri-6/7*, *ergo-1*, *rrf-3*, and *eri-1* [10]. But the *eri-6/7* and *ergo-1* Eri mutants exhibit nearly complete depletion of the 26G siRNAs and a subset of secondary WAGO 22G siRNAs that are produced from these particular primary 26G siRNAs [12,58]. Because SynMuv B mutants exhibit a very strong Eri phenotype that is comparable to that of other well-studied Eri mutants, we investigated the landscape of small RNAs that are generated in these mutants.

**Fig 6:**
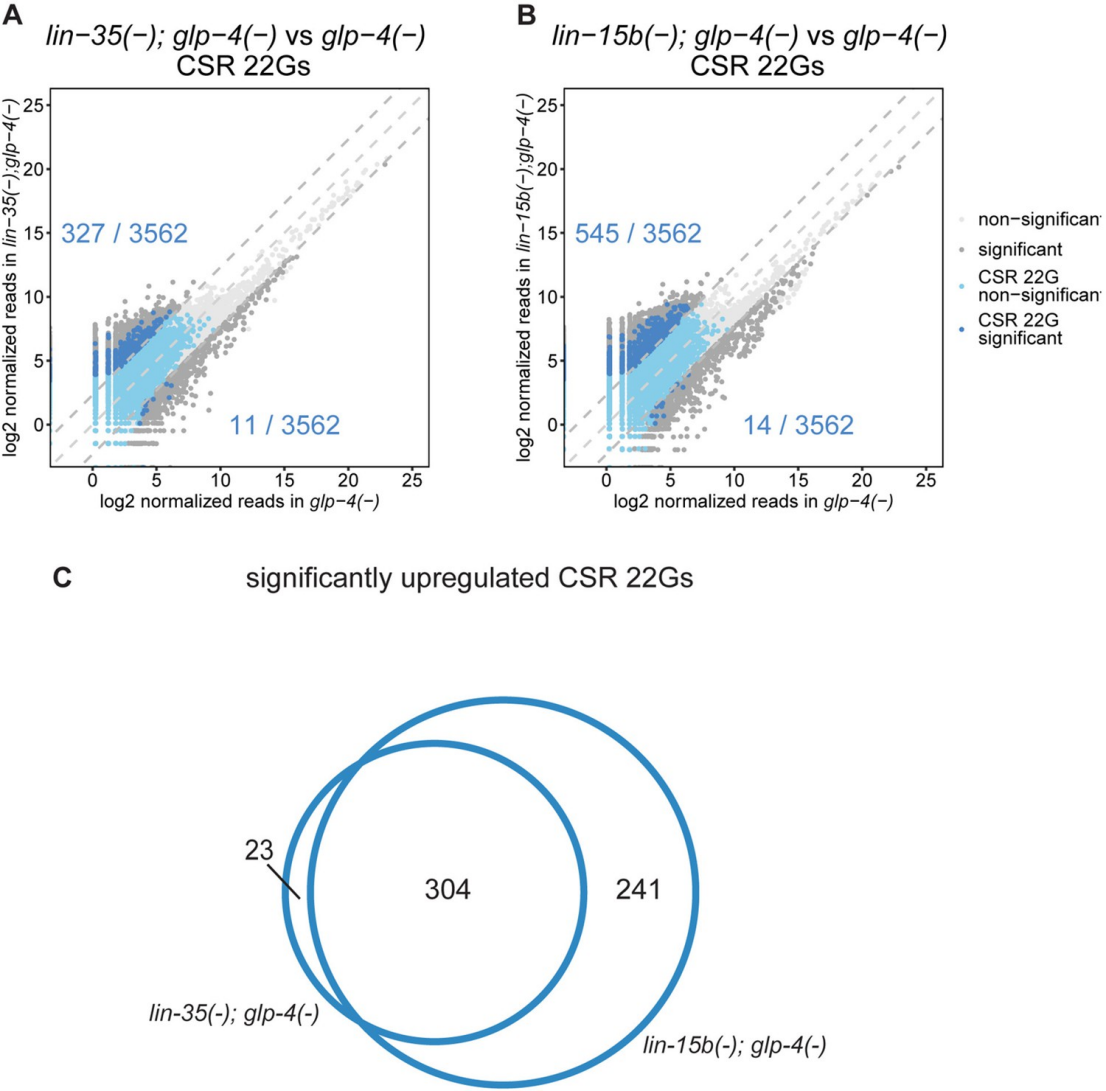
A particular class of CSR 22Gs are up-regulated in synMuv B mutants. (**A, B**) Comparison of normalized reads of CSR 22Gs expressed in *lin-35(n745); glp-4(bn2)* (A) and *lin-15b(n744); glp-4(bn2)* (B) with *glp-4(bn2)*. Dark gray data points represent small RNAs that are differentially expressed by 5-fold and adjusted *p*-value < 0.05. Dark blue data points represent a subset of CSR 22G siRNAs that are significantly up-regulated by atleast 5-fold. Light blue data points represent other CSR 22G siRNAs. The rest of the small RNAs are represented as light gray data points. The lines denoting equal expression, 5-fold higher expression and 5-fold lower expression are shown as gray dashed lines. The dark blue numbers indicate the number of CSR 22Gs that are up-regulated in the synMuv B backround (upper) or the control background (lower). (**C**) Venn diagram representing overlap between CSR 22Gs up-regulated in synMuv B mutants in (A) and (B). The numerical data underlying the graphs shown in this figure can be found in the Supporting information data table named S1 Raw Data.

Given that many of the key synMuv B mutant phenotypes are observed in polyploid somatic cells (the 8C hypoderm for LIN-3 activation and the 32C intestine for ectopic P-granule formation), we first carried out deep sequencing characterization of all siRNAs in synMuv B double mutants that produce little to no germline: *glp-4(bn2)*, *lin-35(n745)*; *glp-4(bn2)* and *lin-15B(n744); glp-4(bn2)* grown at the non-permissive *glp-4* temperature of 25°C (Figs 6, S9, and S10). The *glp-4(bn2)* mutation causes a temperature sensitive genetic ablation of the *C*. *elegans* germline when animals are grown at 25°C. The complete list of the significantly up-regulated and down-regulated by a factor of 5-fold siRNAs are presented in the S1 Data and GEO accession numbers for these data sets can be found in the methods section.

We observed greater than a 5-fold increase in about 10% of the detected CSR-associated 22G siRNAs in both *lin-35(n745)*; *glp-4(bn2)* and *lin-15B(n744); glp-4(bn2)* mutants compared to the *glp-4(bn2)* control animals (Fig 6A and 6B). There was significant overlap between the genes targeted by the up-regulated CSR-22Gs in both *lin-35(n745)*; *glp-4(bn2)* and *lin-15B(n744); glp-4(bn2)* mutants, suggesting that a common pathway is misregulated in these distinct synMuv B mutants (Fig 6C). SynMuv B mutants are also known to misexpress many genes that are normally germline-restricted in somatic tissues such as the intestine [7,29]. Because the animals that we analyzed have no germline, the significant increase in CSR-22G siRNAs that are observed in these synmuvB mutants may reflect the somatic misexpression of these germline genes, perhaps in the intestine. We also observed loss of about half of the detected ERGO-26G siRNA in both *lin-35(n745)*; *glp-4(bn2)* and *lin-15B(n744); glp-4(bn2)* (S9C and S10C Figs). This partial loss of ERGO-26G siRNAs in synmuvB mutants is less severe than the complete depletion of ERGO 26G siRNAs in *eri-1*, *eri-6*, *eri-7*, and *ergo-1* loss of function mutants [12,58]. It is possible that the synMuv B mutants affect 26G siRNA production in the intestine, for example, whereas other *eri-* mutants may affect more cell types.

WAGO-22G siRNAs were differentially expressed in both *lin-35(n745)*; *glp-4(bn2)* and *lin-15B(n744); glp-4(bn2)*, with some showing increased and others showing decreased expression (S9G and S10G Figs). We also observed a decrease in the expression of a few microRNAs and piRNAs in both *lin-35(n745)*; *glp-4(bn2)* and *lin-15B(n744); glp-4(bn2)* (S9K, S9L, S10K, and S10L Figs). An exciting possibility is that these particular miRNAs, for example, *mir-8191* may be located, in locally amplified regions that depend on the dREAM complex, as has been observed for the *Drosophila* chorion genes [36–39]. Finally, we found very little change in the abundance of ALG class 26G siRNAs (S9A and S10A Figs).

Additionally, we carried out in parallel, the deep sequencing of small RNAs in *lin-15B(n744)*, *lin-35(n745)*, *lin-9(n112)*, and *lin-52(n771)* synMuv B mutants with intact germlines (for GEO accession numbers for these data sets, see Methods section). We did not detect any striking differential expression of any of the classes of small RNAs, possibly because the abundance of small RNAs derived from the germline may mask the changes in siRNA expression that may occur in the soma (S11–S14 Figs).

### Molecular epistasis analysis of the enhanced RNAi in synMuv B mutants

The 26G siRNAs in animals are synthesized via the action of the RdRPs RRF-1,2,3 and EGO-1, which produce longer dsRNAs that are further processed into smaller 26G-RNAs through DICER and PIR-1/phosphatase [57–59,62,63]. Our deep sequencing analysis of the synMuv B mutants is consistent with the decline in 26G siRNAs in most Eri mutants, such as *eri-1(mg366*) [58] or *eri-6/7* and *ergo-1* [10,12,64]. To further explore the 26G axis of siRNAs, we mined mRNAseq data sets (from the NCBI Geo-collection) of *lin-35(n745)* and *lin-15B(n744)* mutants, to see whether transcripts of any genes that are known to cause enhanced RNAi (such as *eri-* genes, *rrf-3* etc), when mutant were down-regulated in synMuv B mutants. However, we did not observe lower mRNA levels for any of the known genes that mutate to an enhanced RNAi phenotype in the *lin-15B* and *lin-35* mRNA-seq data sets (see S2–S4 Data files). This suggests that the decrease in 26G siRNAs in the synMuvB mutants is not due to the down-regulation of other known *eri-* genes. 26G-siRNAs are bound by ERGO-1 Argonaute which then targets those siRNAs to particular target mRNAs, approximately 100 recently acquired retroviral elements [10–12]. To test whether synMuv B mutations affect ERGO-1 protein levels or localization to in turn affect 26G siRNA levels, we observed the ERGO-1 protein using a GFP-tagged full-length ERGO-1 protein (*tor147[GFP*::*3XFLAG*::*ergo-1*, the longest predicted isoform [65]. We used RNAi to inactivate synMuv B genes or to inactivate genes that encode suppressors of synMuv B genes, and then observed the localization and expression of *GFP*::*3XFLAG*::ERGO-1 to determine if down-regulating the activity of either synMuv B genes or synMuv Eri suppressor genes can impact the spatiotemporal expression of GFP tagged-*ergo-1* in animals. The GFP tagged-ERGO-1 fusion protein was strongly expressed in the seam cells during the L3 stage and later in the vulval epithelial cells in the mid-L4 and young adult stages (S3A–S3F Fig). We targeted the synMuv B genes *lin-9* and *lin-13* (S3G–S3N Fig), and the synMuv suppressor gene *isw-1* by RNAi (S3O–S3R Fig) and found that the *ergo-1* expression pattern was unchanged. Thus ERGO-1 is unlikely to be a direct or indirect target of synMuv B protein regulation.

### The expression pattern and subcellular localization of *lin-15B* and *lin-35* synMuv B proteins

A detailed description of the subcellular expression/localization patterns for any synMuv B gene product has not been previously reported. We investigated the expression patterns and subcellular localization of the LIN-15B and LIN-35 proteins using *LIN-15B*::*EGFP* and *LIN-35*::*EGFP* protein fusions, both generated by Mihail Sarov via a recombineering pipeline [66]. In these fusion protein reporter strains, the synMuv B gene is tagged by a protein fusion to EGFP at its C terminus in the context of roughly 40 kb of genomic DNA cloned on a fosmid, and then subsequently biolistically transformed at a relatively low copy number [66]. These strains are likely to recapitulate the endogenous expression/localization patterns of their tagged proteins and given their low copy number representation, they may not be subject to the transgene silencing typical of high-copy number transgenes that is commonly associated with Eri-genotypes. These synMuv B GFP fusion proteins were previously used to map the binding sites of the synMuv B proteins on the promoters of genes genome wide [67]. We detected robust LIN-15B::EGFP protein localization in both the hypodermal cells and intestinal cells during the mid-L4 stage and later animals. Intriguingly, LIN-15B::EGFP is localized subnuclearly, forming punctate EGFP foci in the hypodermal and intestinal nuclei. These foci may represent genomic binding sites for client genes of the LIN-15B THAP domain transcription factor, or even more interestingly, given that the dREAM complex mediates genome amplification of chorion genes in *Drosophila*, particular chromosomal regions that are amplified and continue to bind to synMuv B proteins [36]. To quantitate the abundance of LIN-15B::EGFP, we quantitated GFP in particular hypodermal cells and intestinal cells from the anterior, middle, and posterior (S4 Fig). We measured LIN-15B::EGFP fluorescence intensity in these cells using Image J and the number of LIN-15B::EGFP subnuclear foci was manually scored.

These subnuclear foci of LIN-15B do not change under RNAi targeting genes such *mut-16*, *rde-1*, or *rde-4*, which when deactivated impair RNA interference (S5 Fig). The subnuclear foci of LIN-15B also remain unperturbed under RNAi that targets genes known to enhance RNAi such as *eri-6*, *lin-9*, *lin-13*, *lin-35*, *lin-37*, and *lin-52 or* genes that are known to suppress the enhanced response to RNAi of synMuv B mutants such as *isw-1* and *mes-4* (S5 Fig). Crossing lin*-35(n745)* null mutant into the LIN-15B::EGFP reporter strain did not change LIN-15B localization or the average number of subnuclear foci (S6A–S6D Fig). Taken together, these findings suggest that the LIN-15B localization is not coupled to its role to activate the antiviral RNAi response.

Similar observations of the *lin-35*/retinoblastoma expression pattern and protein localization using a lin*-35*::*EGFP* translational fusion gene revealed that LIN-35::EGFP is strongly expressed in all intestinal cells, where it localizes to the nucleus as well as to the nucleolus (S7A and S7B Fig). Each intestinal cell showed 1 or 2 LIN-35::EGFP nucleolar inclusions as well as uniform localization across the nucleus (S7A and S7B Fig). There were no obvious changes to LIN-35 localization or expression level in strains induced to have an enhanced or defective RNAi by inactivation of *eri* or *mutator/rde* genes (S7C–S7E Fig).

## Discussion

The potency of an injected 1 kb dsRNA for *C*. *elegans* RNA interference, discovered by Fire and Mello almost 30 years ago, was the tip of a very large RNAi iceberg. In fact, the complexity of the endogenous small RNA world immediately emerged from the genetic analysis of RNAi: the first RNAi-defective mutants identified included a large set of known mutator genes that had already emerged from searches for mutations that enhance transposition or reversion of transposons; defects in RNA interference was the molecular basis of many mutator mutants that release transposons from RNAi control [1,4,5,7,68,69]. Mutants that disable RNAi in turn activate transposable elements, integrated retroviruses in the *C*. *elegans* genome, the remnants of past successful retroviral infections that are normally silenced by RNAi, which now become active if key RNAi components are defective to then insert or revert from an insertion in any gene [68]. Thus, RNAi is not just a response to *in vitro* produced dsRNAs which are injected, their biological clients are natural endogenous dsRNAs that produce siRNAs which the same genetic pathways mediate their activities.

Similarly, the *C*. *elegans* enhanced RNAi mutants have highlighted a similar physiological regulation of antiviral defense or transposon mobilizations; enhanced RNAi or Eri mutants have emerged from genetic screens at about the same frequency as RNAi defective mutations (Rde, Mut, etc.). Each of the mutant classes, RNAi defective (Rde) and enhanced RNAi (Eri), points to these phenotypes as physiological states, with the mutants frozen in a state of either no RNAi defense or higher than normal RNAi defense. Conversely, *Saccharomyces cerevisiae* jettisoned its RNAi pathway to allow its permanent infection by the killer RNA virus [70].

The synMuv B genes emerged from large genetic and RNAi screens for enhanced RNAi mutants or gene inactivations [7,11], but were discovered 20 years earlier in unrelated comprehensive genetic analysis of receptor tyrosine kinase signaling during *C*. *elegans* vulval development [71,72]. Our analysis of the mRNA expression profiles of synMuv B null mutants showed a significant overlap between genes up-regulated in the synMuv B mutants and those induced upon viral infection by the Orsay RNA virus [41]. Notably, the 100× or more induction of genes in synMuv B mutants that are also strongly induced by Orsay virus, for example, members of the PALS gene family, suggests a potential link between synMuv B mutations and the activation of a coordinated genetic response akin to that observed during viral infections. The dramatic up-regulation of Orsay virus response genes in synMuv B mutants, even in the absence of viral infection, suggested that the down-regulation of synMuv B gene activity could mimic a physiological state of viral resistance, enhanced RNAi, that is normally induced in an actual viral infection, to activate antiviral defense mechanisms, including RNAi. The robust induction of *pals-5p*::*GFP* expression in synMuv B mutants, even in the absence of actual viral infection, suggests that these mutants constitutively activate their antiviral defense pathways.

Because the *C*. *elegans* synMuv B mutants *lin-35* and *lin-15B* activate highly congruent gene expression responses to those of an Orsay RNA virus infection, we tested whether an actual Orsay infection down-regulates synMuv B activity: whether an Orsay virus infection can induce a Muv phenotype in a synMuv A mutant, just as a synMuv B; synMuv A double mutant shows the Muv phenotype. We found that Orsay viral infection of a single synMuv A mutant that never shows the Muv phenotype causes a reproducible 10% penetrant Muv phenotype, but Orsay viral infection of wild-type animals does not cause a Muv phenotype (shown in Fig 5A and 5B). And in support of the model that decreased synMuv B gene activity is part of the normal *C*. *elegans* antiviral response, we found that synMuv B mutants have 50 to 130× lower viral RNA levels during an Orsay virus infection than wild type (shown in Fig 5C). Thus, down-regulation of synMuv B activity to enhance the RNAi is a key component in the defense response to viral infection. Recently, Tecle and colleagues also observed a reduction in viral RNA levels in a different *lin-15B* mutant than what was used in our study [73]. Thus, synMuv B gene activity is integral to defense in an actual viral infection and that viral infection induces a physiological state akin to the enhanced RNAi observed in multiple synMuv B mutants.

Why is the penetrance of the Muv phenotype not higher than 10% during a viral infection of a synMuv A mutant, when null mutations in synMuv B in the background of a null synMuv A mutation can cause 100% penetrant Muv phenotype? The most likely explanation is that a viral infection lowers synMuv B activity but not to zero as in a synMuv B null mutant, that the synMuv B activity decrease during a viral infection is most similar to a non-null synMuv B mutant. In addition, the Muv phenotype of a synMuv A; synMuv B double mutant is caused by the loss of the synMuv B gene specifically in the hypodermis; the loss of synMuv B in the hypodermis is specifically required for the Muv phenotype of a synMuv A; synMuv B double mutant [22]. The loss of synMuv B and synMuv A in the hypodermis causes more than 100× increased expression of the LIN-3 ligand which is secreted to the adjacent affect vulval cells to cause the Muv phenotype in those vulval cells [15]. Because Orsay virus infection induces *pals-5p*::*GFP* in the intestine, the primary tissue where the virus infection is thought to occur via its ingestion [42], it is possible that synMuv B down-regulation in the hypodermis during a viral infection of the intestine is far less than a synMuv B null mutant, thus the lower penetrance of the Muv phenotype.

We also explored the link between gene inactivations that suppress the enhanced RNAi in synMuv B mutants and their activation of antiviral response genes. RNAi inhibition of known suppressors of the enhanced RNAi phenotype of synMuv B genes, such as *isw-1* and *mes-4*, caused a significant reduction in *pals-5p*::*GFP* induction by a synMuv B mutation, indicating a potential interplay between RNAi and antiviral defense pathways regulated by synMuv B genes. Interestingly, our findings suggest that the Eri phenotype induced by synMuv B mutations may engage distinct pathways from other known Eri mutants, such as mutations in the RNA dependent RNA polymerase *rrf-3* or the *eri-1* exonuclease, highlighting the complexity of antiviral defense mechanisms in *C*. *elegans*. The observed suppression of *pals-5p*::*GFP* expression upon *isw-1* and *mes-4* knockdown in synMuv B mutants under non-infection conditions underscores the role of these genes in modulating the antiviral response. However, inactivation of *isw-1* or *mes-4* did not suppress the induction of *pals-5p*::*GFP* during an actual viral infection, suggesting that synMuv B induction of the *pals-5p*::*GFP* response, may be one of several pathways that regulates the expression of *pals-5* during an actual viral infection.

*C*. *elegans* RNAi pathways surveil thousands of genomic loci in wild-type *C*. *elegans* under non-viral infection conditions to produce hundreds to thousands of siRNAs per million reads. Most of this massive cloud of siRNAs against their own genes are missing in animals with defects in RNAi, most dramatically in the Mutator mutants [30]. In contrast, null mutations in the enhanced RNAi mutant *eri-6/7*, encoding an RNA helicase, disrupts the production of a far more circumscribed set of siRNAs that silence approximately 100 integrated retroviral elements [10–13]. This Eri mutant may be enhanced for RNAi because the desilenced retroviral elements trigger ER stress, probably due to the replication of viral sequences in the ER, and the enhanced RNAi of an actual or falsely sensed infection [10]. Our survey of the small RNA landscape in synMuv B Eri mutants showed a 5-fold increase in 10% CSR-class 22G siRNAs, 5-fold decrease in 50% of ERGO-class 26G siRNAs, 7% of WAGO-class 22G siRNAs, 4% of piRNAs, and 14% microRNAs in synMuv B mutants. The partial loss of ERGO-26Gs and piRNAs in synMuv B mutants is distinct from the previously characterized *eri* mutants [12,58]. One model for the molecular basis of enhanced RNAi mutants is that there is a limiting component to RNA interference; for example, the silencing of transposons uses some limiting factor for RNAi such that if the silencing of retrotransposons is disabled, that limiting factor is released for the dsRNA-programmed silencing of feeding RNAi [64]. However, the differences in the small RNA landscape of the synMuv B mutants suggest a distinct mechanism may underlie the enhanced RNAi of synMuv B mutants: the targets of ERGO-class 26Gs, piRNAs and WAGO-class 22Gs include pseudogenes, foreign elements and transposons, loss of these small RNAs in synmuvB mutants may cause the misexpression of these genes and may explain the constitutively active anti-viral readiness of the synmuvB mutants. It is possible that the de-repression of integrated viruses, the activation of transposons, in the synMuv B mutants in fact causes a viral activation to in turn induced stronger RNAi. The induction of enhanced RNAi during a viral infection makes excellent biological sense.

The synMuv B genes encode homologues of the dREAM complex, which are conserved across animals and plants, including the LIN-35/retinoblastoma (Rb) tumor suppressor, and the Rb complex components LIN-53 /RbAp48, LIN-37/MIP40, LIN-54/MIP120, DPL-1 /DP, LIN-9/MIP130 [16,19–21]. The synMuv A genes *lin-8*, *lin-56*, and *lin-15A* encode widely expressed *C*. *elegans* nuclear proteins with no clear homologues outside of nematodes [17,18], and in the case of *lin-8*, there exists more than a dozen paralogues in *C*. *elegans* and many dozen in *C*. *japonicum*, located in clusters of duplicated paralogues [46]. The nuclear localization of the synMuv A proteins is consistent with their strong genetic interaction with the nuclearly localized synMuv B dREAM complex proteins, but nothing in the anonymous synMuv A protein sequences suggests a mechanism of action.

While the analysis of the synMuv A and synMuv B mutants in specification of the ventral precursor cells has been extensive and beautiful, how it connects to the enhanced RNAi of the synMuv B mutants is not instantly obvious from the beautiful molecular genetic analysis of LIN-3 signaling [15,16,20,21,23]. The molecular basis of how the DNA-binding proteins and associated proteins of the synMuv B pathway actually intersect with the PIWI proteins and RdRPs that are central to RNAi has not been characterized. Our investigations suggested that RNAi suppression of the Eri-phenotype of synMuv B mutants by *isw-1* or *mes-4* RNAi do not affect the localization of LIN-15B or LIN-35 (see S5 and S7 Figs). This suggests that a molecular mechanism distinct from the localization or abundance of the synMuv B proteins regulates the intensity of the RNAi pathway.

However, extensive analysis of the many synMuv B orthologues in *Drosophila* has revealed their activity in endoreduplication of particular client genes, and the detailed analysis of *C*. *elegans* synMuv B genes in the vulval cell signaling pathway has revealed an analogous intersection with endoreduplication. The *Drosophila* dREAM complex binds specifically at replication origins that flank the *Drosophila* chorion genes to control their local (about 50 kb) endoreduplication to nearly 100× the gene dosage of the normally diploid *Drosophila* genome [36]. This enables the regulated production of the abundant eggshell proteins; mutations in the dREAM complex cause a humpty dumpty phenotype, fragile eggs due to insufficient production of chorion proteins [38,74]. The dREAM complex proteins bind directly to these chorion gene replication origins and are necessary for the localized amplification of just those genes; in the *Drosophila* retinoblastoma mutant, unregulated endoreduplication occurs [37–39].

This intersection of the dREAM complex with endoreduplication is also a hallmark of the synMuv B gene function in LIN-3 growth factor signaling from the polyploid *C*. *elegans* hypodermis to the adjacent vulval cells that show the Muv phenotype in the synMuv B mutants. The polyploid hypodermal cells hyp7 cell is the focus of synMuv B gene function in *C*. *elegans* for the regulation of LIN-3 production [15]. The *C*. *elegans* hypoderm is normally endoreduplicated to 8C (3 full genome duplications) or more at adult stages [75].

Analysis of other shared *C*. *elegans* synMuv B mutant phenotypes also suggests that they also function in another polyploid tissue, the intestine, to control the production of P-granules which are approximately 1 micron ribonucleoprotein granules intimately connected to RNAi [30]. The P-granules are normally germline-specific multi-protein and RNAs that assemble into the originally described LLPS particles of the single blastomere that is the precursor of the *C*. *elegans* germline [28]. Loss of function mutations in multiple *C*. *elegans* synMuv B genes cause misexpression of these normally germline-specific P-granules in somatic tissues, most dramatically in the polyploid intestine and hypodermis [7,29]. The adult *C*. *elegans* intestine is normally endoreduplicated from diploid at hatching to 32C (5 inferred full genome duplications with the DAPI-light microscope DNA quantitation), with one duplication at each larval stage but no chromosome condensation or mitosis [25]. The hyp7 hypoderm where ectopic P granules are also observed is 8C. The P granules, as macromolecular complexes that are visible at light microscope magnification, are filled with abundant proteins and RNAs, which like chorion proteins, represent candidate genes for localized endoreduplication [29,31–34,76].

It may be significant that nearly all of the 50 genes that are strongly up-regulated in synMuv B mutants and during viral infection are clustered in the genome (see S1 Fig and the S5 Data). For example, the *pals* genes as well as the *lin-8*-like genes (*lido-genes*) localize to genomic clusters with several *pals* or *lido* genes within cluster. The clusters are also very plastic in *Caenorhabditis* evolution, varying down to just 3 *lido* genes in *C*. *remaniae* and up to 58 *lido* genes in *C*. *japonicum*. These clusters of poorly conserved *pals* and *lido* genes may be under dREAM complex control of their local amplification, which may serve to increase the gene copy number for example in the intestine.

That RNAi is highly regulated is not surprising: RNAi has evolved by selection for potent antiviral defense during almost a billion years of eukaryotic infection by viruses. But it is surprising that the dREAM complex is so central to RNAi. And the mammalian-conserved synMuv B pathway reveals new pathways to activate production or response to small interfering RNAs and may be targeted with drugs to activate human antiviral responses or responses to RNAi-based pharmaceuticals.

## Materials and methods

### Nematode strains used

**Strains made available through the CAENORHABDITIS GENETICS CENTER (CGC):** *N2 (wild-type)*, *lin-35(n745)*, *lin-52(n771)*, *lin-9(n112)*, *lin-15B(n744)*, *lin-15AB(n765)*, *eri-6/7(mg441)*, *eri-1(mg366)*, *NL2099 [rrf-3(pk1426]*, *ERT54 [jyIs8 [pals-5p*::*GFP; myo-2p*::*mCherry]*, *mgIs30 [rol-6(su1006)*::*lin-14 3′-UTR*, *col-10*::*lacZ*, *lim-6*::*gfp])*, *OP184[wgIs184 [lin-15B*::*TY1*::*EGFP*::*3xFLAG + unc-119(+)]*, *OP763[wgIs763 [lin-35*::*TY1*::*EGFP*::*3xFLAG + unc-119(+)]*, *lin-8(n2731)*, *qIs56(lag-2*::*gfp*, *unc-119(þ)*, *eri-1(mg366)*, *eri-6(mg379)*, *rrf-3(pk1426)*, *mut-16(pk710)*.

More details regarding the gene mutations featured here can be found at http://www.wormbase.org.

**Strains generated in our lab for this study:** *lin-35(n745) glp-4(bn2)*, *glp-4(bn2); lin-15B(n744)*, *glp-4(bn2)*, *lin-35(n745); jyIs8*, *lin-52(n771); jyIs8*, lin-15B(W485*), lin-15B(W485*); jj2284, lin-15B(W485*);FAS207, *eri-1(mg366); jyIs8*, *eri-6(mg379); jyIs8*, *rrf-3(pk1426); jyIs8*, *lin-9(n112); qIs56*, *lin-35(n745); qIs56*.

Strains sent by others*: *FAS207*, *unc-119(ed3) III; zuIs261[ges-1(5′ UTR)*::*npp-9*::*mCherry*::*BLRP*::*3xFLAG*::*npp-9(3’UTR); unc-119(+)]; zuIs236[his-72(5’ UTR)*::*BIRA*::*GFP*::*his-72(3’ UTR); unc-119(+)]*.

JJ2284 *unc-119(ed3) III; zuIs261[ges-1(5’ UTR)*::*npp-9*::*mCherry*::*BLRP*::*3xFLAG*::*npp-9(3’ UTR); unc-119(+)*. *These strains were kindly provided by Steven Henikoff and Florian Steiner.

Generating the strain lin-15B(W485*): In brief, gravid animals were injected with a CRISPR/Cas9 mix containing 30 pmol *S*. *pyrogenes* Cas9 (IDT), 90 pmol tracrRNA (IDT), 95 pmol crRNA oHG6, 2.2 mg repair template oHG8, and 80 ng of *PRF4*::*rol-6 (su1006)* plasmid. The F1 progeny that exhibited the *rol* phenotype as well as their siblings were singled and genotyped for the edit using primers oHG9 and oHG10, followed by a restriction digest using AseI (New England Biolabs).

### Oligos used

oHG6: AGAATGAGAATTCGAATCAT

oHG8: GAGCGGCCCGAAGAGAATGAGAATTCGAATCATTAATGGGGAATGCGTCAGACGGATATGGAATTCTTGGC

oHG9: ACAAAAACTTTTATCAAGATATTTCGATCCTG

oHG10: ACAAAAACTTTTATCAAGATATTTCGATCCTG

**Counting intestinal nuclei:** Animals infected with Orsay virus were collected in M9, crushed and filtered through a 0.22 μm filter; 50 μl of the resulting lysate was used to infect animals. Equal volume of M9 was used for the mock infection. Four L4 animals were infected with mock or Orsay virus lysate. Animals were allowed to propagate for 2 generations. When the animals were running out of food, the animals were chunked to fresh plates pre-infected with the Orsay virus lysate. F2 adults were picked into sodium azide on an agarose pad and imaged on the Zeiss Axio Imager Z.1 within an hour. The number of mCherry expressing intestinal nuclei was scored using the 40× objective.

**RT-qPCR for Orsay RNA1: Growth conditions:** Ten 60 mm plates of *mut-16(pk710)* animals infected with Orsay virus and the OP50 lawn that they were grown on were collected in approximately 8.5 ml of M9. Worms were spun down and the supernatant was moved to a fresh tube. The worms were moved to a 1.5 ml microcentrifuge tube and crushed using a pestle. The worm debris was spun down and the resulting clear lysate was added to the previously collected supernatant. The pooled supernatant was filtered through a 0.22 μm filter and 350 μl of the resulting filtrate was used to infect approximately 20 fresh 60 mm plates each. Equal volume of M9 was used for mock infection. Adults of the relevant genotypes were bleached using sodium hypochlorite treatment and embryos were allowed to hatch on the infected lawns. Approximately 1,200 animals were collected in Trizol 3 days after plating as adults (N2 and *eri-1*) or late L4s (*mut-16*, *lin-15b*, *lin-35*).

**RNA extraction and RT-qPCR:** Samples were freeze–thawed thrice using liquid nitrogen. After the addition of 1-bromo-3-chloropropane, the RNA in the aqueous phase was precipitated by incubating with isopropanol for 2 h at −30°C. Samples were centrifuged at 21,000 × g for 30 min at 4°C to pellet the RNA. The pellet was washed thrice in 70% ethanol, dried and resuspended in water; 500 ng of RNA was used to make cDNA with BIO-RAD iScript cDNA Synthesis Kit (cat# 1708890) according to manufacturer’s instructions, and 2 μl of the resulting cDNA was used for Quantitative PCR with BIO-RAD iQ SYBR Green Supermix (cat# 170–8880). The primers used for amplifying *eft-2* and Orsay RNA1 are listed at the end of this section. The cycle numbers for Orsay RNA1 were normalized to the respective cycle numbers for *eft-2*. Two biological replicates with 2 technical replicates were done. The values were all normalized to the average of the 4 values for the wild-type N2 infected samples. Two-tailed Student’s unpaired *t* test was done to evaluate *p*-values.

### Oligos used

oHG17: ACGCTCGTGATGAGTTCAAG [qPCR FP for *eft-2*]

oHG18: ATTTGGTCCAGTTCCGTCTG [qPCR RP for *eft-2*]

oHG53: ACCTCACAACTGCCATCTACA [qPCR FP for Orsay RNA1 [48]]

oHG50: GACGCTTCCAAGATTGGTATTGGT [qPCR RP for Orsay RNA1 [48]]

### siRNA Sequencing: Growth conditions

Eggs were hatched and arrested as synchronous L1 larvae and then plated on *E*. *coli* OP50 and grown at either 20°C for 78 to 96 h (*glp-4+*) or 25°C for 72 to 74 h (*glp-4-*). Samples were collected for RNA isolation when most animals had reached adult stage, which varied between strains. There was some stage asynchrony within the SynMuv mutant strains because of differences in development timing.

**RNA isolation**: RNA was isolated from whole animals using Trizol and chloroform extraction according to the manufacturer’s recommendations but with the addition of a second chloroform extraction step (Life Technologies, cat# 15596018).

**Small RNA sequencing**: Total RNA was treated with RppH to reduce small RNA 5′ triphosphates to monophosphates following the manufacturer’s recommendations (New England Biolabs, cat# M0356S) [77]; 16 to 30-nt small RNAs were size selected on a 17% polyacrylamide/urea gel and purified using the ZR small-RNA PAGE Recovery Kit (Zymo Research, cat# R1070). Small RNA sequencing libraries were prepared using the NEBNext Multiplex Small RNA Library Prep Set for Illumina following the manufacturer’s recommendations but with the 3′ ligation step changed to 16°C for 18 h to improve capture of methylated small RNAs (New England Biolabs, cat# E7300S). PCR products corresponding in size to adapter-ligated small RNAs were size selected on a 10% polyacrylamide non-denaturing gel, electrophoretically transferred to DE81 chromatography paper, eluted at 70°C for 20 min in the presence of 1 M NaCl, and precipitated at −80°C overnight in the presence of 13 μg/ml glycogen and 67% EtOH. Small RNA read processing, including adapter trimming, quality filtering, mapping, and counting, was done with tinyRNA v1.5 using the default configuration [55]. Reference sequences and annotations were based on the *C*. *elegans* WS279 release [78].

### GEO accession numbers and genotype information for the siRNAseq data

**Project accession number:** GSE283983

**Individual sample accession numbers:** *GSM8675299 (N2 rep 1)*, *GSM8675300 (N2 rep 2)*, *GSM8675301(lin-15b rep 1)*, *GSM8675302 (lin-15b rep 2)*,*GSM8675303 (lin-35 rep 1)*, *GSM8675304 (lin-35 rep 2)*, *GSM8675305 (lin-52 rep 1)*, *GSM8675306 (lin-52 rep 2)*, *GSM8675307 (lin-9 rep 1)*, *GSM8675308 (lin-9 rep 2)*, *GSM8675309 (glp-4 rep 1)*, *GSM8675310 (glp-4 rep 2)*, *GSM8675311 (lin-15b glp-4 rep 1)*, *GSM8675312 (lin-15b glp-4 rep 2)*, *GSM8675313 (lin-35 glp-4 rep 1)*, *GSM8675314 (lin-35 glp-4 rep 2)*.

### Generating the top 100 up and down list of siRNA targets

- DESeq2 analysis using R was performed on 3 sample groups at once (i.e., *glp-4; lin-15B* and *lin-35 glp-4* and *glp-4*) for the 0 mm (mismatch) set.
- Pairwise comparisons (i.e., *lin-15B; glp-4* and *glp-4* OR *lin-35; glp-4* and *glp-4*) yielded log2FoldChange and adjusted *p*-values (padj). To identify the siRNAs with significant changes up in the synMuv B mutants, the list of siRNA targets was filtered as follows:
  ○ Greater than or equal to 5-fold change (i.e., log2FoldChange = log2(5) = 2.32);
  ○ Less than or equal to adjusted *p*-value of 0.05; and
  ○ Greater than or equal to 10 counts of normalized reads in the sy*nMuv B; glp-4* strains.
- To identify the siRNAs with significant changes down in the synMuv B mutants, the list of siRNA targets was filtered as follows:
  ○ Less than or equal to 0.2-fold change (i.e., log2FoldChange = log2(0.2) = −2.32);
  ○ Less than or equal to adjusted *p*-value of 0.05; and
  ○ Greater than or equal to 10 counts of normalized reads in the *glp-4* strains.
- Each of the lists of siRNA targets were arranged in the order of greatest fold change (i.e., greatest log2FoldChange for siRNA targets that are the most up-regulated and lowest log2FoldChange for siRNA targets that are the most down-regulated).

### mRNA sequencing analysis

Fastq files were downloaded from GEO (GEO Accession numbers GSE62833 and GSE155190). The STAR aligner [79] was used to map sequencing reads to transcriptome in the ce10 reference genome. Read counts for individual genes were produced using the count function in HTSeq [80], followed by the estimation of expression values and detection of differentially expressed transcripts using EdgeR [81], which included only the genes with count per million reads (CPM) > 1 for 2 or more samples. Differentially expressed genes were defined by the cutoffs of > log2 fold change and false discovery rate (FDR) < 0.05. Venn diagram in S1 Fig was generated using venn and matplotlib in Python 3.

### GEO accession numbers and genotype information

GSM4697084 (N2 starved L1-rep1), GSM4697085 (N2 starved L1-rep2), GSM4697089 [lin-35(n745) starved L1-rep1)], GSM4697090 (lin-35(n745) starved L1-rep2), GSM4697102 (lin-15B(n744) starved L1-rep1), GSM4697105 (lin-15B(n744) starved L1-rep2), GSM1534086 (lin-35[JA1507(n745) L3-rep1), GSM1534087(lin-35[JA1507(n745) L3-rep2).

## Supporting information

S1 Fig Gene expression analysis reveals antiviral defense genes that are up-regulated in synMuv B mutants. Most genes (80/91) up-regulated during Orsay virus infection of wild-type animals [41] are also up-regulated in *synMuvB* mutants [82,83]. Venn diagram representing overlap between the published data sets is on the left; 46 genes that are up-regulated in all datasets is listed on the right. All genes analyzed here were up-regulated by at least 5-fold compared to the respective control samples. The **S5 Data** shows that most of the up-regulated genes this analysis revealed are clustered in the genome, indicative of local regulation of gene expression or perhaps even gene endoreduplication by synMuv B genes.
(TIF)

S2 Fig Ectopic *lag-2p*::*GFP* expression in the intestinal cells upon virus-infection remains unaltered upon removal of *isw-1* activity by RNAi. (A) Fluorescent micrographs showing 2 *lag-2p*::*GFP* expressing animals that are infected with the Orsay virus raised on *E*. *coli* that express double stranded RNA against either empty vector (control) or the synMuv suppressor gene *isw-1*. Expression of *lag-2p*::*GFP* within the distal tip cells is indicated by yellow arrow heads and the ectopic fluorescence of *lag-2p*::*GFP* in the intestine is also indicated. (B) Quantification of the fluorescence intensity of *lag-2p*::*GFP* within the intestine of animals that are infected with the Orsay virus raised on *E*. *coli* that express double stranded RNA against either the L4440 empty vector (labeled as control) or *isw-1* is shown. A schematic of the general orientation of the worm is shown above the image panels. Scale bar is indicated. The raw microscopy images shown in this figure have been deposited in Zenodo and are accessible at DOI: 10.5281/zenodo.14289232.
(TIF)

S3 Fig Spatiotemporal expression pattern of ERGO-1 remains unperturbed by the loss of function of synMuv B genes. (A–F) Brightfield and GFP channel micrographs depicting the temporal expression pattern of *ergo-1*::*GFP* from a transgenic strain that expresses a full-length ERGO-1 PIWI protein fused at its C terminus to GFP. (G–R) *ergo-1*::*GFP* expression after synMuv B (*lin-9 or lin-13*), or Muv suppressor (*isw-1*) RNAi. Developmental stage is labeled. Scale bar is indicated. The raw microscopy images shown in this figure have been deposited in Zenodo and are accessible at DOI: 10.5281/zenodo.14289232.
(TIF)

S4 Fig LIN-15B C-terminal protein fusion::EGFP is localized to subnuclear foci in *C*. *elegans* polyploid hypodermal and intestinal cells. (A–E) Brightfield and EGFP micrographs of LIN-15B localization in representative hypodermal (panels A and B), intestinal nuclei (panels C–E). Abbreviations for the different intestinal cells that are boxed are provided below the image panels. Scale bar is indicated. The raw microscopy images shown in this figure have been deposited in Zenodo and are accessible at DOI: 10.5281/zenodo.14289232.
(TIF)

S5 Fig Quantification of LIN-15B::EGFP fluorescence intensity and number of subnuclear foci. (A, B) Quantification of EGFP fluorescence and number of subnuclear LIN-15B foci in 3 representative hypodermal cells for synMuv B (*lin-9*, *lin-13*, *lin-35*, *lin-37 and lin-52*), Eri associated-gene (*eri-6*), synMuv suppressors (*mes-4*, *isw-1*), RNAi defective genes (*rde-1*, *rde-4*, *mut-16*) under RNAi conditions. (C, D) Quantification of EGFP fluorescence and number of subnuclear LIN-15B foci within 6 representative intestinal cells for synMuv B (*lin-9*, *lin-13*, *lin-35*, *lin-37 and lin-52*), Eri associated-gene (*eri-6*), synMuv suppressors (*mes-4*, *isw-1*), RNAi defective genes (*rde-1*, *rde-4*, *mut-16*) under RNAi conditions. In the graphs shown in C and D the abbreviations AD refers to anterior dorsal cell, AV refers to anterior ventral cell, MA refers to midgut anterior cell and MP refers to midgut posterior cell, PD refers to posterior gut dorsal cell and PV refers to posterior gut ventral cell. The numerical data underlying the graphs shown in this figure can be found in the Supporting information data table named S1 Raw Data.
(TIF)

S6 Fig Quantification of LIN-15B::EGFP fusion protein fluorescence intensity and number of subnuclear foci in *lin-35(n745)* null mutants. (A, B) EGFP fluorescence and number of subnuclear LIN-15B foci within 3 representative hypodermal cells under RNAi conditions targeting L4440 vector (control), RNAi defective genes (*rde-1*, *mut-16*), and Muv suppressor (*isw-1 and mes-4*). (C, D) Intensity of EGFP fluorescence and number of subnuclear LIN-15B foci within 6 representative intestinal cells where the abbreviations AD refers to anterior dorsal cell, AV refers to anterior ventral cell, MA refers to midgut anterior cell and MP refers to midgut posterior cell, PD refers to posterior gut dorsal cell, and PV refers to posterior gut ventral cell. RNAi was performed targeting L4440 vector (control), RNAi defective genes (*rde-1*, *mut-16*), and Muv suppressor genes (*isw-1 and mes-4*). These experiments were carried out in a *lin-35(n745)* null background. The numerical data underlying the graphs shown in this figure can be found in the Supporting information data table named S1 Raw Data.
(TIF)

S7 Fig LIN-35::EGFP localization in the nucleolus of *C*. *elegans* intestinal cells. (A, B) Fluorescent micrographs of LIN-35::EGFP fusion protein localization in the intestinal nucleoli of wild-type animals. Magnification used to visualize the cells is indicated. The image shown in panel B are the same cells that are boxed in panel A, visualized under a higher magnification. (C) Fluorescent micrographs of LIN-35::EGFP localization within the intestinal cells (boxed in panel A), of the F1 progeny of worms raised on *E*. *coli* expressing double stranded RNA against either empty vector (l4440) or various synMuv B genes (*lin-9*, *lin-13*, *lin-15b*, *lin-37*, *lin-52*, *lin-54*, *lin-61*, *tam-1*, *hpl-1*, and *dpl-1*) or a synMuv A gene (*lin-8*) is shown. Arrow heads indicate the presence of a nucleolar inclusion of LIN-35::EGFP. (D) Quantification of the number of LIN-35::EGFP nucleolar inclusions that are seen in animals raised on *E*. *coli* expressing double stranded RNA against either empty vector (l4440) or various synMuv B genes (*lin-9*, *lin-13*, *lin-15b*, *lin-37*, *lin-52*, *lin-54*, *lin-61*, *tam-1*, *hpl-1*, and *dpl-1*) or a synMuv A gene (*lin-8*) is shown. (E) Quantification of the number of LIN-35::EGFP nucleolar inclusions that are seen in animals raised on *E*. *coli* expressing double stranded RNA against either empty vector (l4440) or various genes that are critical for the worms ability to perform RNAi. Scale bar is indicated. The raw microscopy images shown in this figure have been deposited in Zenodo and are accessible at DOI: 10.5281/zenodo.14289232.
(TIF)

S8 Fig *lin-15b(-)* animals exhibit altered intestinal nuclear morphology. Top panel shows wild-type *JJ2284* animals under no virus and Orsay virus-infected conditions. Lower panel shows a second independent line of lin-15b(W485*); JJ2284 animals under no virus and Orsay virus-infected conditions. White arrows indicate elongated intestinal nuclei that are elongated. Scale bar is indicated. The raw microscopy images shown in this figure have been deposited in Zenodo and are accessible at DOI: 10.5281/zenodo.14289232.
(TIF)

S9 Fig Comparisons of normalized reads of indicated classes of small RNAs in *lin-35(n745); glp-4(bn2)* with *glp-4(bn2)*. Dark gray data points represent small RNAs that are differentially expressed by 5-fold and adjusted *p*-value < 0.05. Colored data points represent indicated classes of small RNAs. The rest of the small RNAs are represented as light gray data points. Lines denoting equal, 5-fold increased, and 5-fold decreased expression are shown as light gray dashed lines. The numbers indicate the number of small RNAs up-regulated in *lin-35(n745); glp-4(bn2)* (upper) and *glp-4(bn2)* (lower). The numerical data underlying the graphs shown in this figure can be found in the Supporting information data table named S1 Raw Data.
(TIF)

S10 Fig Comparisons of normalized reads of indicated classes of small RNAs in *lin-15b(n744); glp-4(bn2)* with *glp-4(bn2)*. Dark gray data points represent small RNAs that are differentially expressed by 5-fold and adjusted *p*-value < 0.05. Colored data points represent indicated classes of small RNAs. The rest of the small RNAs are represented as light gray data points. Lines denoting equal, 5-fold increased and 5-fold decreased expression are shown as light gray dashed lines. The numbers indicate the number of small RNAs upregulated in *lin-15b(n744); glp-4(bn2)* (upper) and *glp-4(bn2)* (lower). The numerical data underlying the graphs shown in this figure can be found in the Supporting information data table named S1 Raw Data.
(TIF)

S11 Fig Comparisons of normalized reads of indicated classes of small RNAs in *lin-35(n745)* with wild type. Dark gray data points represent small RNAs that are differentially expressed by 5-fold and adjusted *p*-value < 0.05. Colored data points represent indicated classes of small RNAs. The rest of the small RNAs are represented as light gray data points. Lines denoting equal, 5-fold increased and 5-fold decreased expression are shown as light gray dashed lines. The numbers indicate the number of small RNAs up-regulated in *lin-35(n745)* (upper) and wild type (lower). The numerical data underlying the graphs shown in this figure can be found in the Supporting information data table named S1 Raw Data.
(TIF)

S12 Fig Comparisons of normalized reads of indicated classes of small RNAs in *lin-15b(n744)* with wild type. Dark gray data points represent small RNAs that are differentially expressed by 5-fold and adjusted *p*-value < 0.05. Colored data points represent indicated classes of small RNAs. The rest of the small RNAs are represented as light gray data points. Lines denoting equal, 5-fold increased and 5-fold decreased expression are shown as light gray dashed lines. The numbers indicate the number of small RNAs up-regulated in *lin-15b(n744)* (upper) and wild type (lower). The numerical data underlying the graphs shown in this figure can be found in the Supporting information data table named S1 Raw Data.
(TIF)

S13 Fig Comparisons of normalized reads of indicated classes of small RNAs in *lin-9(n112)* with wild type. B Dark gray data points represent small RNAs that are differentially expressed by 5-fold and adjusted *p*-value < 0.05. Colored data points represent indicated classes of small RNAs. The rest of the small RNAs are represented as light gray data points. Lines denoting equal, 5-fold increased and 5-fold decreased expression are shown as light gray dashed lines. The numbers indicate the number of small RNAs up-regulated in *lin-9(n112)* (upper) and wild type (lower). The numerical data underlying the graphs shown in this figure can be found in the Supporting information data table named S1 Raw Data.
(TIF)

S14 Fig Comparisons of normalized reads of indicated classes of small RNAs in *lin-52(n771)* with wild type. Dark gray data points represent small RNAs that are differentially expressed by 5-fold and adjusted *p*-value < 0.05. Colored data points represent indicated classes of small RNAs. The rest of the small RNAs are represented as light gray data points. Lines denoting equal, 5-fold increased and 5-fold decreased expression are shown as light gray dashed lines. The numbers indicate the number of small RNAs up-regulated in *lin-52(n771)*(upper) and wild type (lower). The numerical data underlying the graphs shown in this figure can be found in the Supporting information data table named S1 Raw Data.
(TIF)

S1 Table Top 50 up-regulated genes in *lin-15B(n744*), mutant in starved L1 animals. The 50 most highly up-regulated genes in *lin-15(n744)* mutants during the L1 stage. The log2 Fold-change and the corresponding false discovery rate (FDR), value for each gene that is listed is shown. The gene expression data was derived from our analysis of mRNA seq data sets *[GSM4697084- N2—Rep 1/L1*, *GSM4697085- N2—Rep 2/L1 against GSM4697102- lin-15b(n744)- rep1/L1*, *GSM4697105- lin-15b(n744)- rep2/L1*] that were obtained from the NCBI Geo collection.
(XLSX)

S2 Table Top 50 up-regulated genes in *lin-35(n745*), mutant in starved L1 animals. The 50 most highly up-regulated genes in *lin-35(n745)* mutants during the L1 stage. The log2 Fold-change and the corresponding false discovery rate (FDR), value for each gene that is listed is shown. The gene expression data was derived from our analysis of mRNA seq data sets *GSM4697089- lin-35[JA1507(n745) rep1/L1*, *GSM4697090- lin-35[JA1507(n745) rep1/L1]* that were obtained from the NCBI Geo collection.
(XLSX)

S3 Table Top 50 up-regulated Genes in *lin-35(n745*), mutant at the L3 Stage. The top 50 most highly up-regulated genes in *lin-35(n745)* mutants during the L3 stage. The log2 Fold-change and the corresponding false discovery rate (FDR), value for each gene that is listed is shown. [*GSM1534084- N2-rep1 L3*, *GSM1534085- N2-rep2 compared against GSM1534086- lin-35[JA1507(n745) rep1 L3 and GSM1534087- lin-35[JA1507(n745) rep1 L3]* that were obtained from the NCBI Geo collection.
(XLSX)

S1 Data List of genes to which differentially regulated small RNAs map in *lin-35(n745); glp-4(bn2)* and *lin-15b(n744); glp-4(bn2)* in comparison with *glp-4(bn2)*. The siRNAs that are either Up or Down by a minimum factor of a 5-fold difference in the *lin-15(n744)* and *lin-35(n745)* mutant backgrounds are presented. See the labeled tabs for the genotype information.
(XLSX)

S2 Data Gene expression changes in *lin-35(n745)* starved L1 animals. Shown here are the lists of significant gene expression changes via mRNA seq that are observed in the *lin-35(n745)* synMuv B mutant animals at the L1 stage. The data shown here was obtained from the NCBI geo collection, *GSM4697089 [lin-35(n745) starved L1-rep1)]*, *GSM4697090 (lin-35(n745) starved L1-rep2)]*.
(XLS)

S3 Data Gene expression changes in *lin-35(n745)* starved L3-stage animals. Shown here are the lists of significant gene expression changes via mRNA seq that are observed in the *lin-35(n745)* synMuv B mutant animals at the L3-stage. The data shown here was obtained from the NCBI geo collection, *GSM1534086 (lin-35[JA1507(n745) L3-rep1)*, *GSM1534087(lin-35[JA1507(n745) L3-rep2)]*.
(XLS)

S4 Data Gene expression changes in *lin-15(n744)* starved L1-stage animals. Shown here are the lists of significant gene expression changes via mRNA seq that are observed in the *lin-15(n744)* synMuv B mutant animals at the L1-stage. The data shown here was obtained from the NCBI geo collection, *GSM4697102 (lin-15B(n744) starved L1-rep1)*, *GSM4697105 (lin-15B(n744) starved L1-rep2)]*.
(XLS)

S5 Data Genome location of the top 48 up-regulated genes in synMuv B mutants as well as Orsay virus infected wild type map to a few clusters in the genome. (XLSX)

S1 Raw Data This table contains all the raw numerical values underlying the data presented in both main and supplemental figures, including individual replicates, means, and standard deviation values, as used in the analyses for this study. (XLSX)

## Abbreviations

dsRNA: double-stranded RNA
FDR: false discovery rate
RdRP: RNA-dependent RNA polymerase
RNAi: RNA interference
