# Supplementary figures and images for: Decreased SynMuv B gene activity in response to viral infection leads to activation of the antiviral RNAi pathway in C. elegans
Source: PLoS Biol. 2025 Jan 29;23(1):e3002748. doi: 10.1371/journal.pbio.3002748 (PMC11778786; doi:10.1371/journal.pbio.3002748)

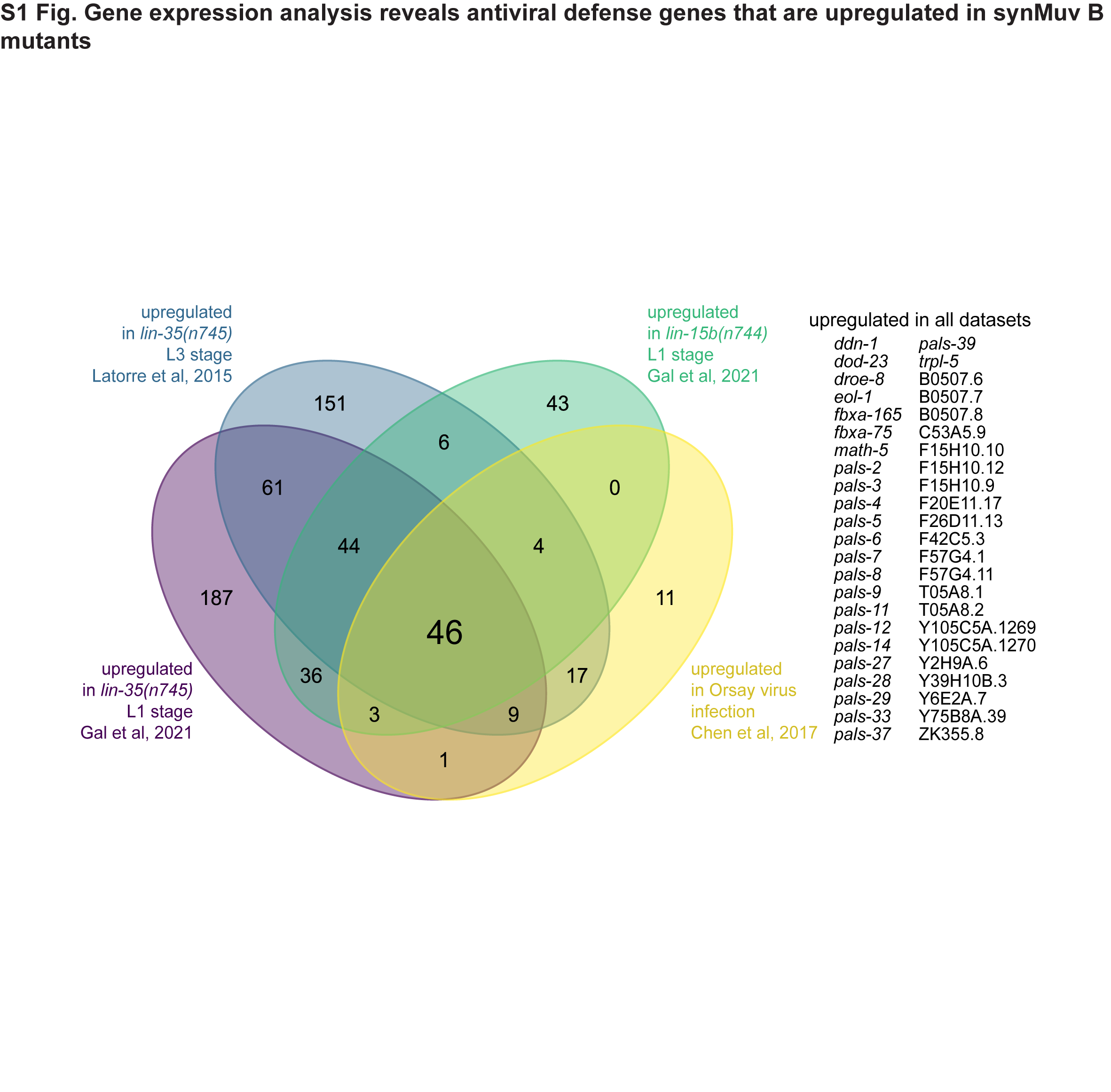

Supplement: S1 Fig — Most genes (80/91) up-regulated during Orsay virus infection of wild-type animals [41] are also up-regulated in synMuvB mutants [82,83]. Venn diagram representing overlap between the published data sets is on the left; 46 genes that are up-regulated in all datasets is listed on the right. All genes analyzed here were up-regulated by at least 5-fold compared to the respective control samples. The S5 Data shows that most of the up-regulated genes this analysis revealed are clustered in the genome, indicative of local regulation of gene expression or perhaps even gene endoreduplication by synMuv B genes. (TIF) [file pbio.3002748.s001.tif]

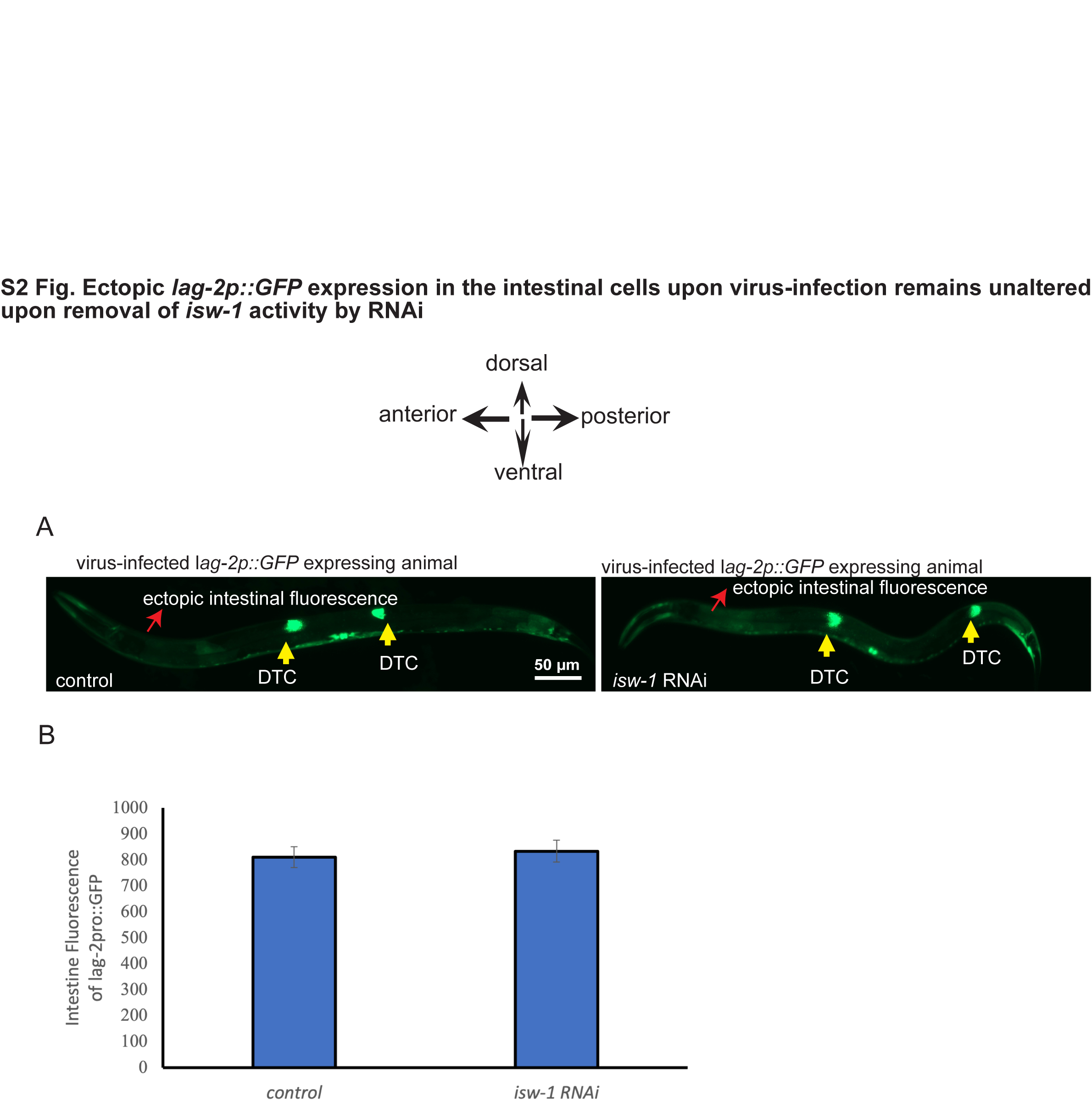

Supplement: S2 Fig — (A) Fluorescent micrographs showing 2 lag-2p::GFP expressing animals that are infected with the Orsay virus raised on E. coli that express double stranded RNA against either empty vector (control) or the synMuv suppressor gene isw-1. Expression of lag-2p::GFP within the distal tip cells is indicated by yellow arrow heads and the ectopic fluorescence of lag-2p::GFP in the intestine is also indicated. (B) Quantification of the fluorescence intensity of lag-2p::GFP within the intestine of animals that are infected with the Orsay virus raised on E. coli that express double stranded RNA against either the L4440 empty vector (labeled as control) or isw-1 is shown. A schematic of the general orientation of the worm is shown above the image panels. Scale bar is indicated. The raw microscopy images shown in this figure have been deposited in Zenodo and are accessible at DOI: 10.5281/zenodo.14289232. (TIF) [file pbio.3002748.s002.tif]

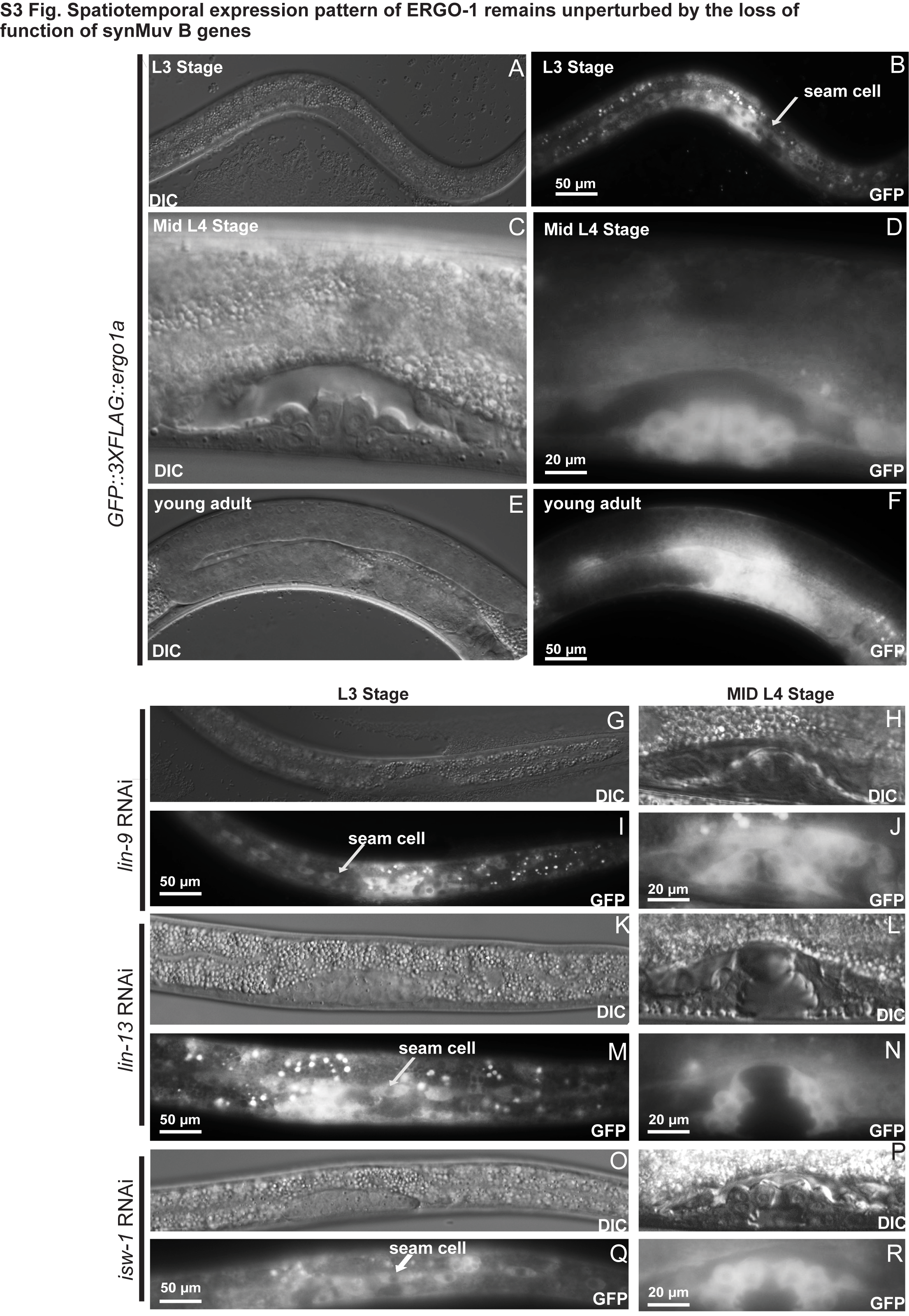

Supplement: S3 Fig — (A–F) Brightfield and GFP channel micrographs depicting the temporal expression pattern of ergo-1::GFP from a transgenic strain that expresses a full-length ERGO-1 PIWI protein fused at its C terminus to GFP. (G–R) ergo-1::GFP expression after synMuv B (lin-9 or lin-13), or Muv suppressor (isw-1) RNAi. Developmental stage is labeled. Scale bar is indicated. The raw microscopy images shown in this figure have been deposited in Zenodo and are accessible at DOI: 10.5281/zenodo.14289232. (TIF) [file pbio.3002748.s003.tif]

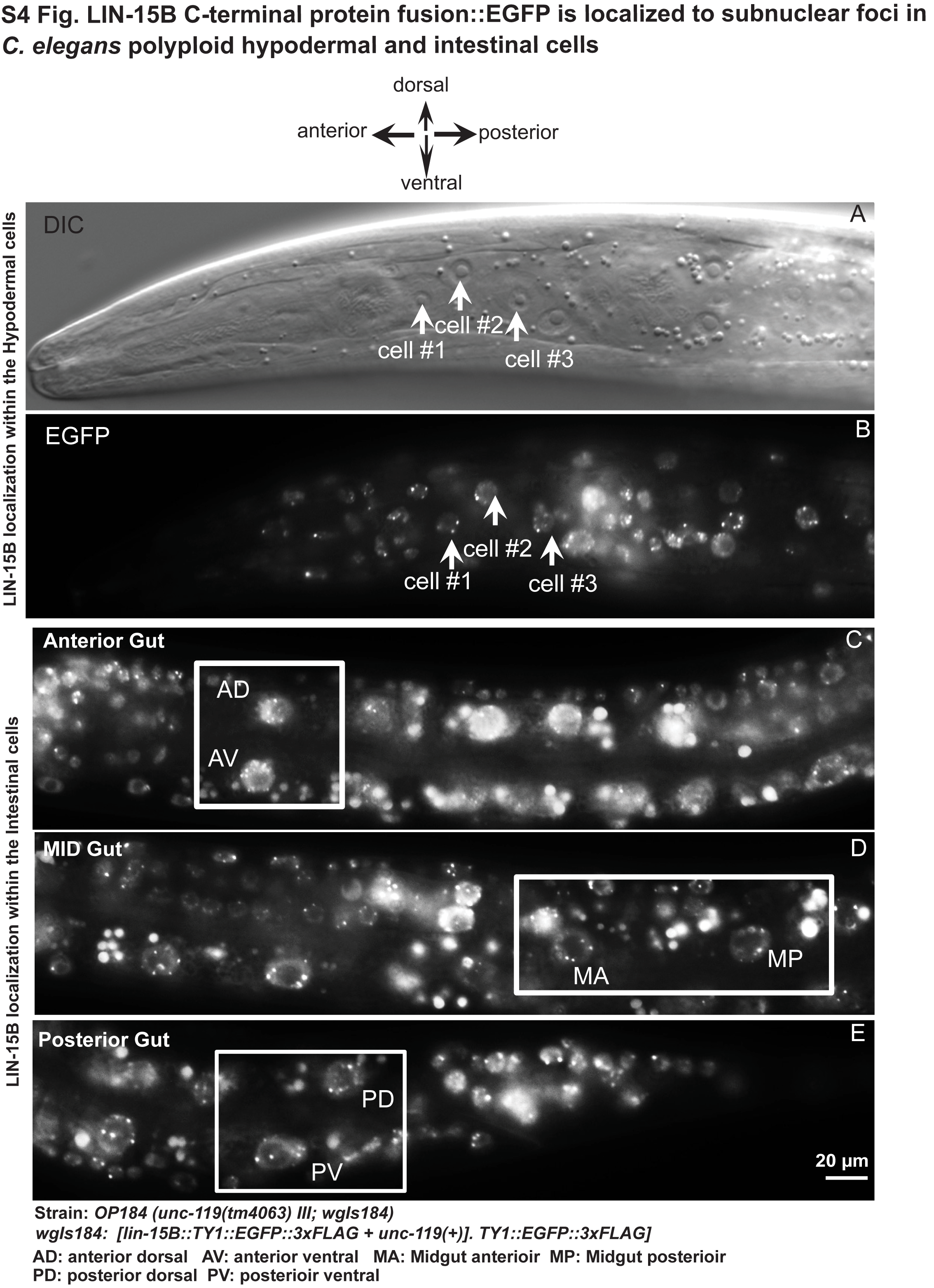

Supplement: S4 Fig — (A–E) Brightfield and EGFP micrographs of LIN-15B localization in representative hypodermal (panels A and B), intestinal nuclei (panels C–E). Abbreviations for the different intestinal cells that are boxed are provided below the image panels. Scale bar is indicated. The raw microscopy images shown in this figure have been deposited in Zenodo and are accessible at DOI: 10.5281/zenodo.14289232. (TIF) [file pbio.3002748.s004.tif]

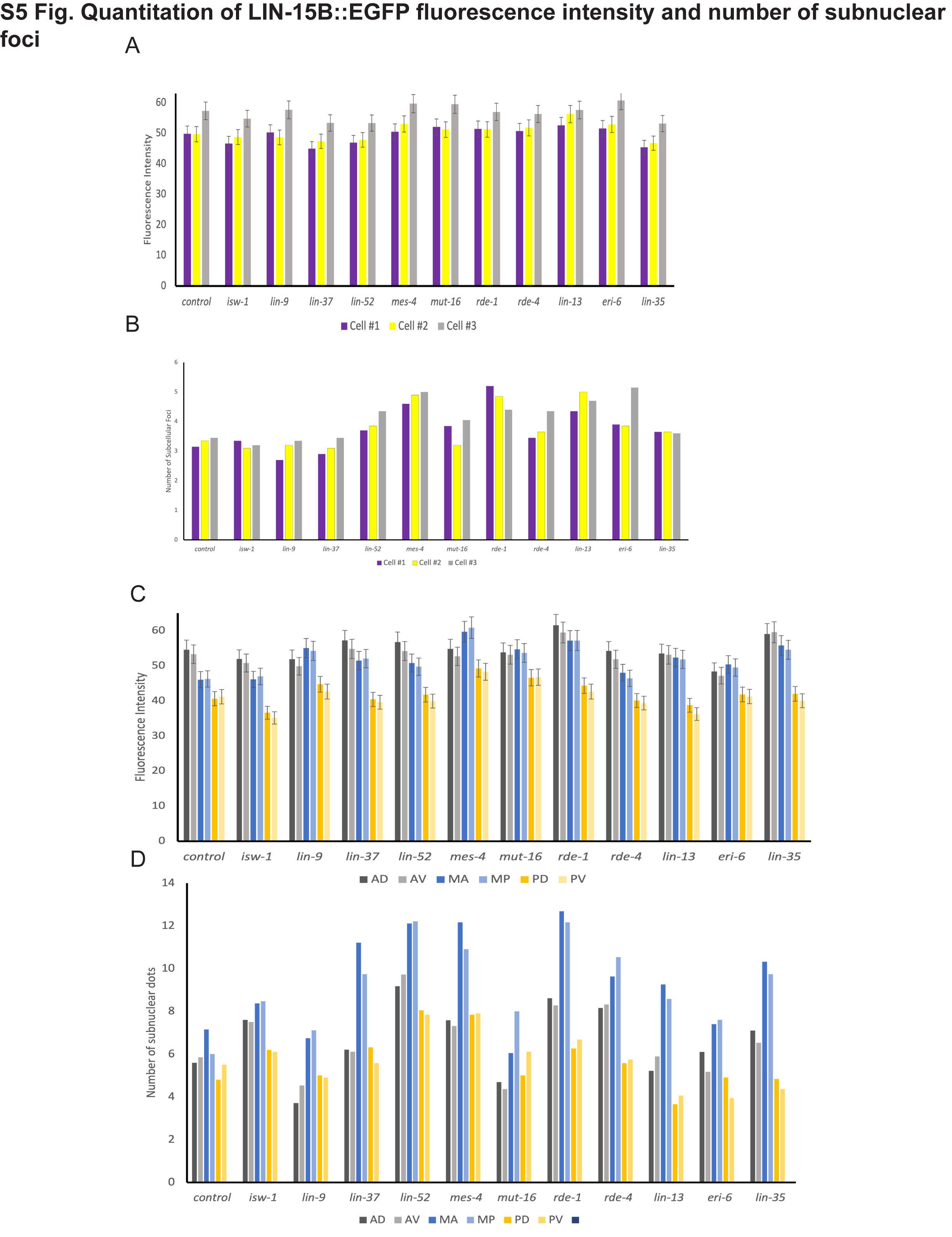

Supplement: S5 Fig — (A, B) Quantification of EGFP fluorescence and number of subnuclear LIN-15B foci in 3 representative hypodermal cells for synMuv B (lin-9, lin-13, lin-35, lin-37 and lin-52), Eri associated-gene (eri-6), synMuv suppressors (mes-4, isw-1), RNAi defective genes (rde-1, rde-4, mut-16) under RNAi conditions. (C, D) Quantification of EGFP fluorescence and number of subnuclear LIN-15B foci within 6 representative intestinal cells for synMuv B (lin-9, lin-13, lin-35, lin-37 and lin-52), Eri associated-gene (eri-6), synMuv suppressors (mes-4, isw-1), RNAi defective genes (rde-1, rde-4, mut-16) under RNAi conditions. In the graphs shown in C and D the abbreviations AD refers to anterior dorsal cell, AV refers to anterior ventral cell, MA refers to midgut anterior cell and MP refers to midgut posterior cell, PD refers to posterior gut dorsal cell and PV refers to posterior gut ventral cell. The numerical data underlying the graphs shown in this figure can be found in the Supporting information data table named S1 Raw Data. (TIF) [file pbio.3002748.s005.tif]

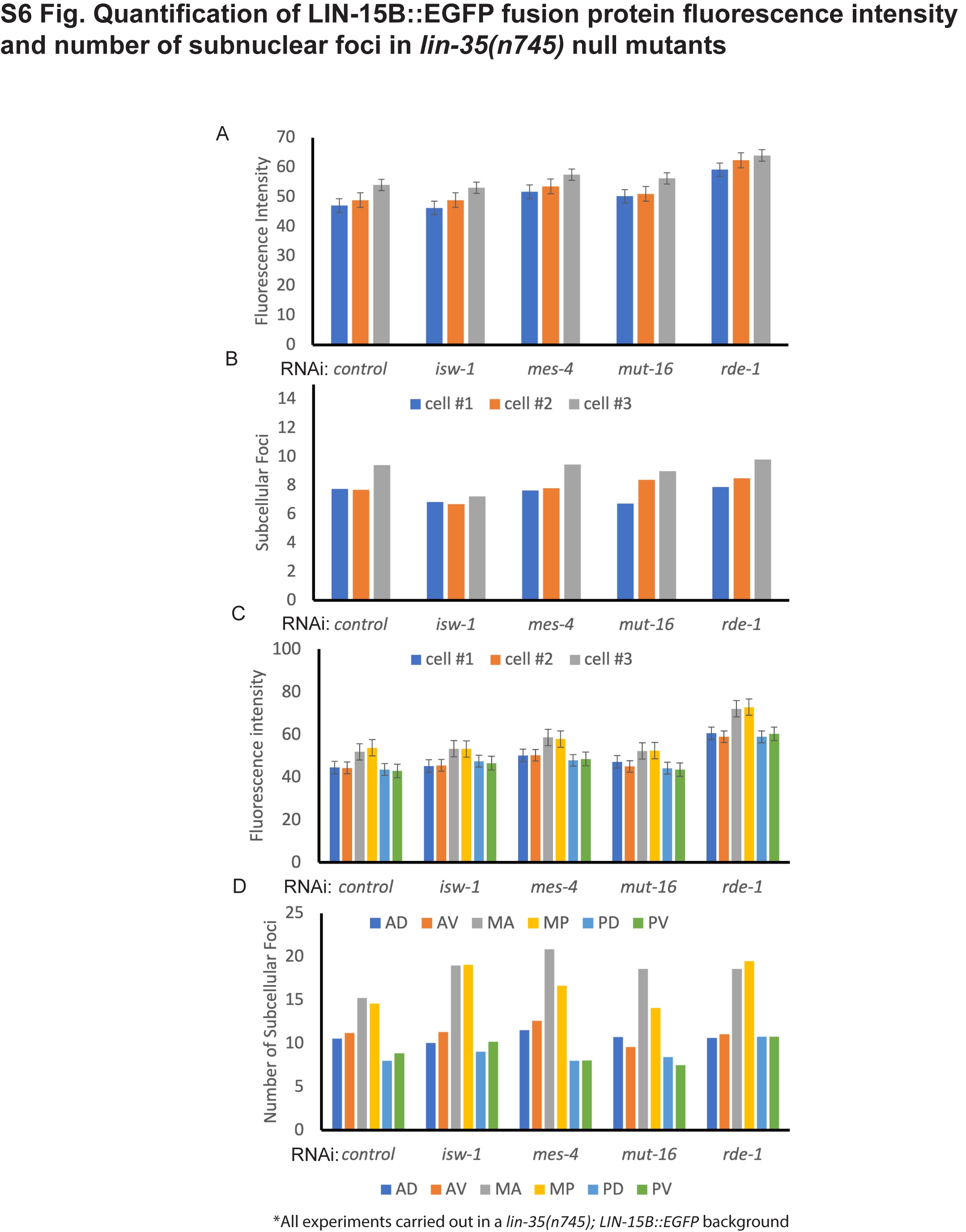

Supplement: S6 Fig — (A, B) EGFP fluorescence and number of subnuclear LIN-15B foci within 3 representative hypodermal cells under RNAi conditions targeting L4440 vector (control), RNAi defective genes (rde-1, mut-16), and Muv suppressor (isw-1 and mes-4). (C, D) Intensity of EGFP fluorescence and number of subnuclear LIN-15B foci within 6 representative intestinal cells where the abbreviations AD refers to anterior dorsal cell, AV refers to anterior ventral cell, MA refers to midgut anterior cell and MP refers to midgut posterior cell, PD refers to posterior gut dorsal cell, and PV refers to posterior gut ventral cell. RNAi was performed targeting L4440 vector (control), RNAi defective genes (rde-1, mut-16), and Muv suppressor genes (isw-1 and mes-4). These experiments were carried out in a lin-35(n745) null background. The numerical data underlying the graphs shown in this figure can be found in the Supporting information data table named S1 Raw Data. (TIF) [file pbio.3002748.s006.tif]

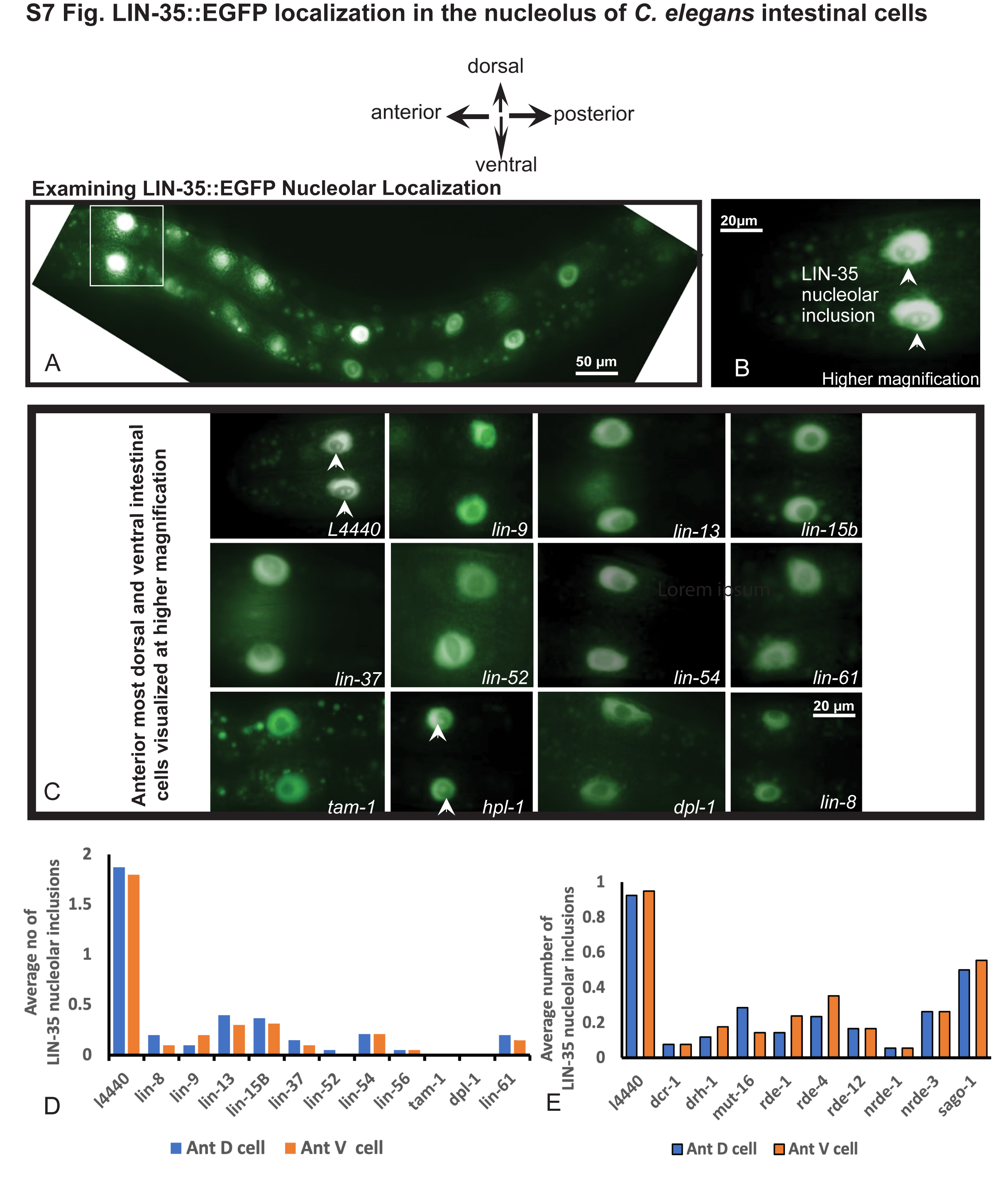

Supplement: S7 Fig — (A, B) Fluorescent micrographs of LIN-35::EGFP fusion protein localization in the intestinal nucleoli of wild-type animals. Magnification used to visualize the cells is indicated. The image shown in panel B are the same cells that are boxed in panel A, visualized under a higher magnification. (C) Fluorescent micrographs of LIN-35::EGFP localization within the intestinal cells (boxed in panel A), of the F1 progeny of worms raised on E. coli expressing double stranded RNA against either empty vector (l4440) or various synMuv B genes (lin-9, lin-13, lin-15b, lin-37, lin-52, lin-54, lin-61, tam-1, hpl-1, and dpl-1) or a synMuv A gene (lin-8) is shown. Arrow heads indicate the presence of a nucleolar inclusion of LIN-35::EGFP. (D) Quantification of the number of LIN-35::EGFP nucleolar inclusions that are seen in animals raised on E. coli expressing double stranded RNA against either empty vector (l4440) or various synMuv B genes (lin-9, lin-13, lin-15b, lin-37, lin-52, lin-54, lin-61, tam-1, hpl-1, and dpl-1) or a synMuv A gene (lin-8) is shown. (E) Quantification of the number of LIN-35::EGFP nucleolar inclusions that are seen in animals raised on E. coli expressing double stranded RNA against either empty vector (l4440) or various genes that are critical for the worms ability to perform RNAi. Scale bar is indicated. The raw microscopy images shown in this figure have been deposited in Zenodo and are accessible at DOI: 10.5281/zenodo.14289232. (TIF) [file pbio.3002748.s007.tif]

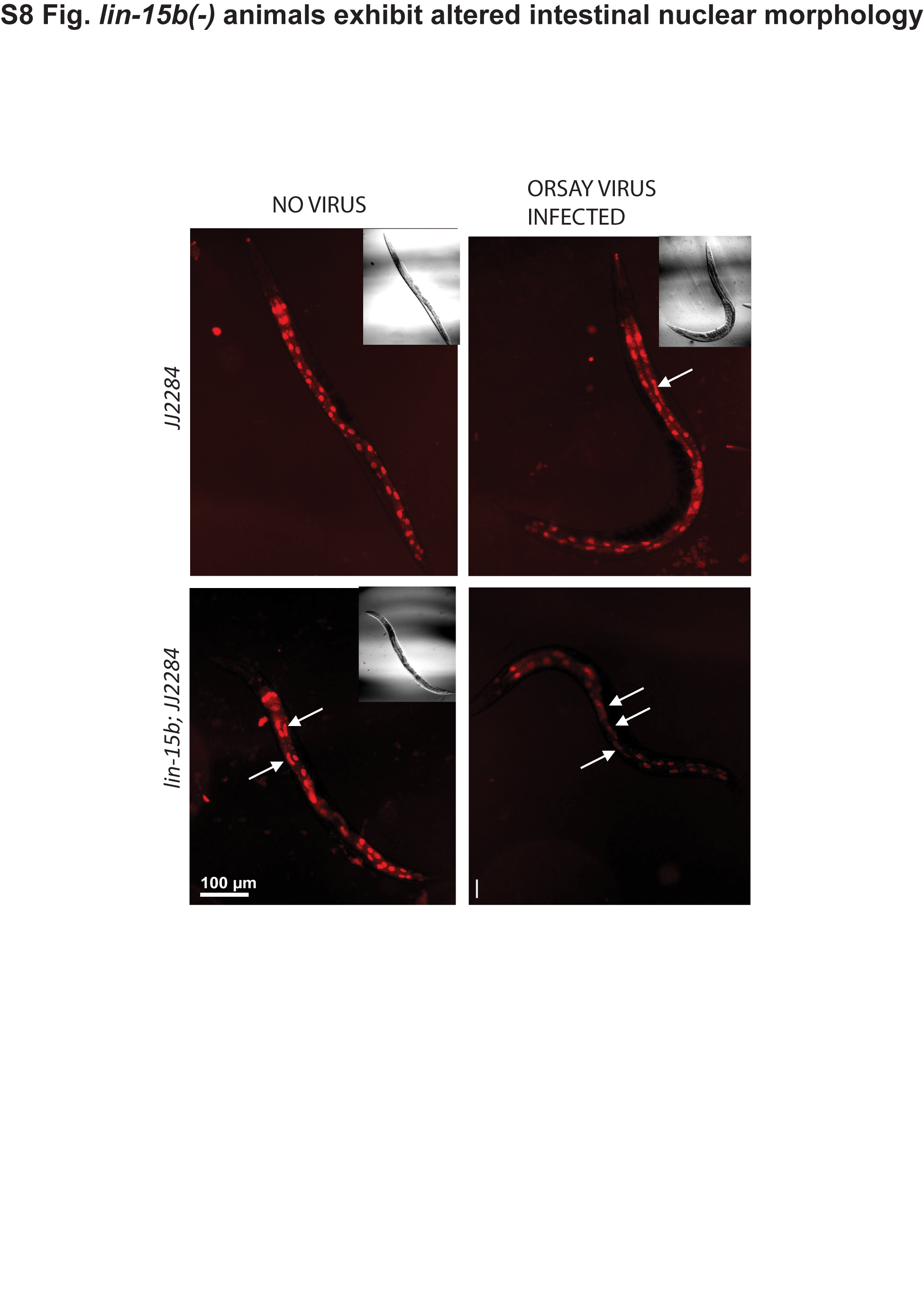

Supplement: S8 Fig — Top panel shows wild-type JJ2284 animals under no virus and Orsay virus-infected conditions. Lower panel shows a second independent line of lin-15b(W485*); JJ2284 animals under no virus and Orsay virus-infected conditions. White arrows indicate elongated intestinal nuclei that are elongated. Scale bar is indicated. The raw microscopy images shown in this figure have been deposited in Zenodo and are accessible at DOI: 10.5281/zenodo.14289232. (TIF) [file pbio.3002748.s008.tif]

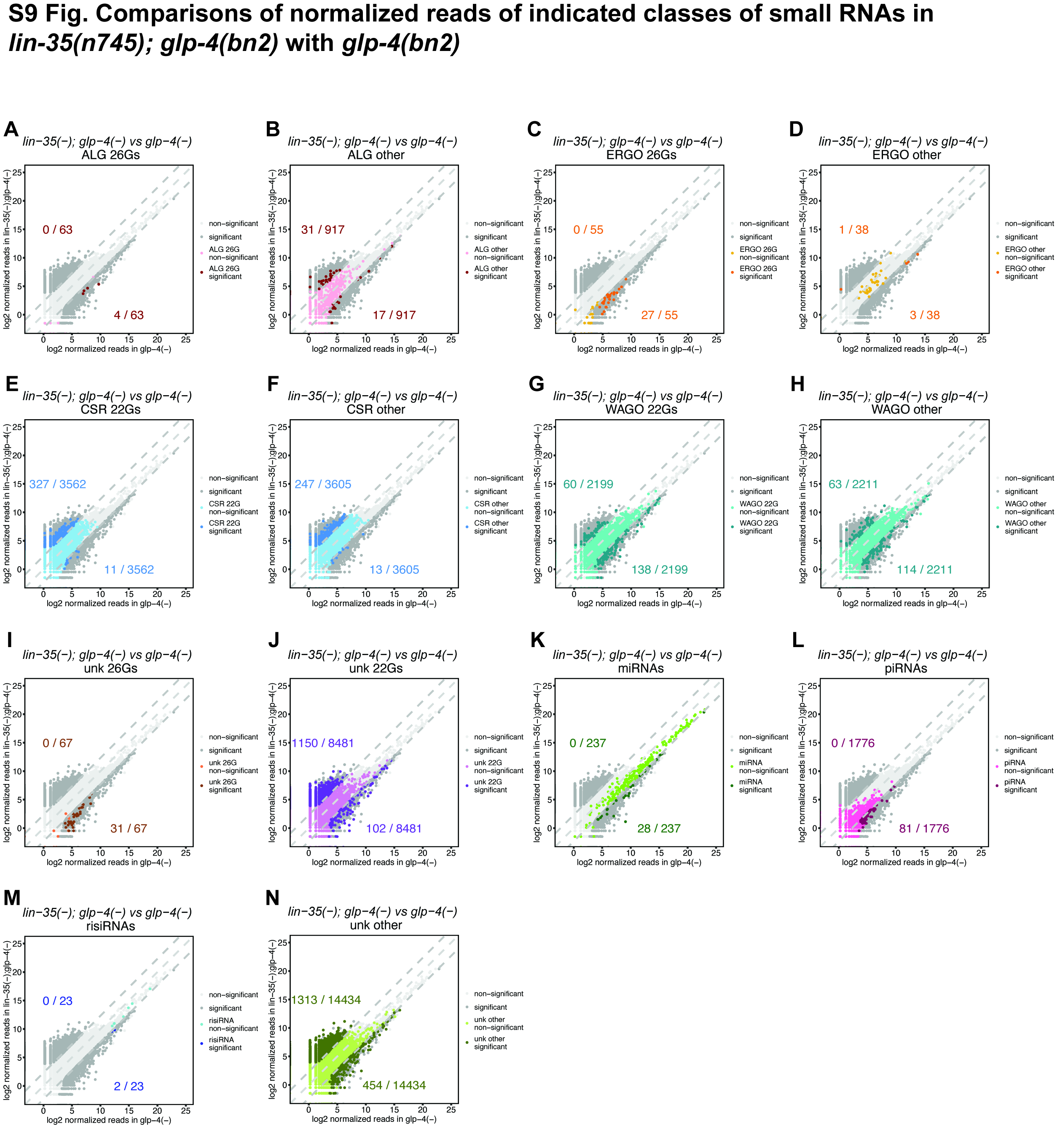

Supplement: S9 Fig — Dark gray data points represent small RNAs that are differentially expressed by 5-fold and adjusted p-value < 0.05. Colored data points represent indicated classes of small RNAs. The rest of the small RNAs are represented as light gray data points. Lines denoting equal, 5-fold increased, and 5-fold decreased expression are shown as light gray dashed lines. The numbers indicate the number of small RNAs up-regulated in lin-35(n745); glp-4(bn2) (upper) and glp-4(bn2) (lower). The numerical data underlying the graphs shown in this figure can be found in the Supporting information data table named S1 Raw Data. (TIF) [file pbio.3002748.s009.tif]

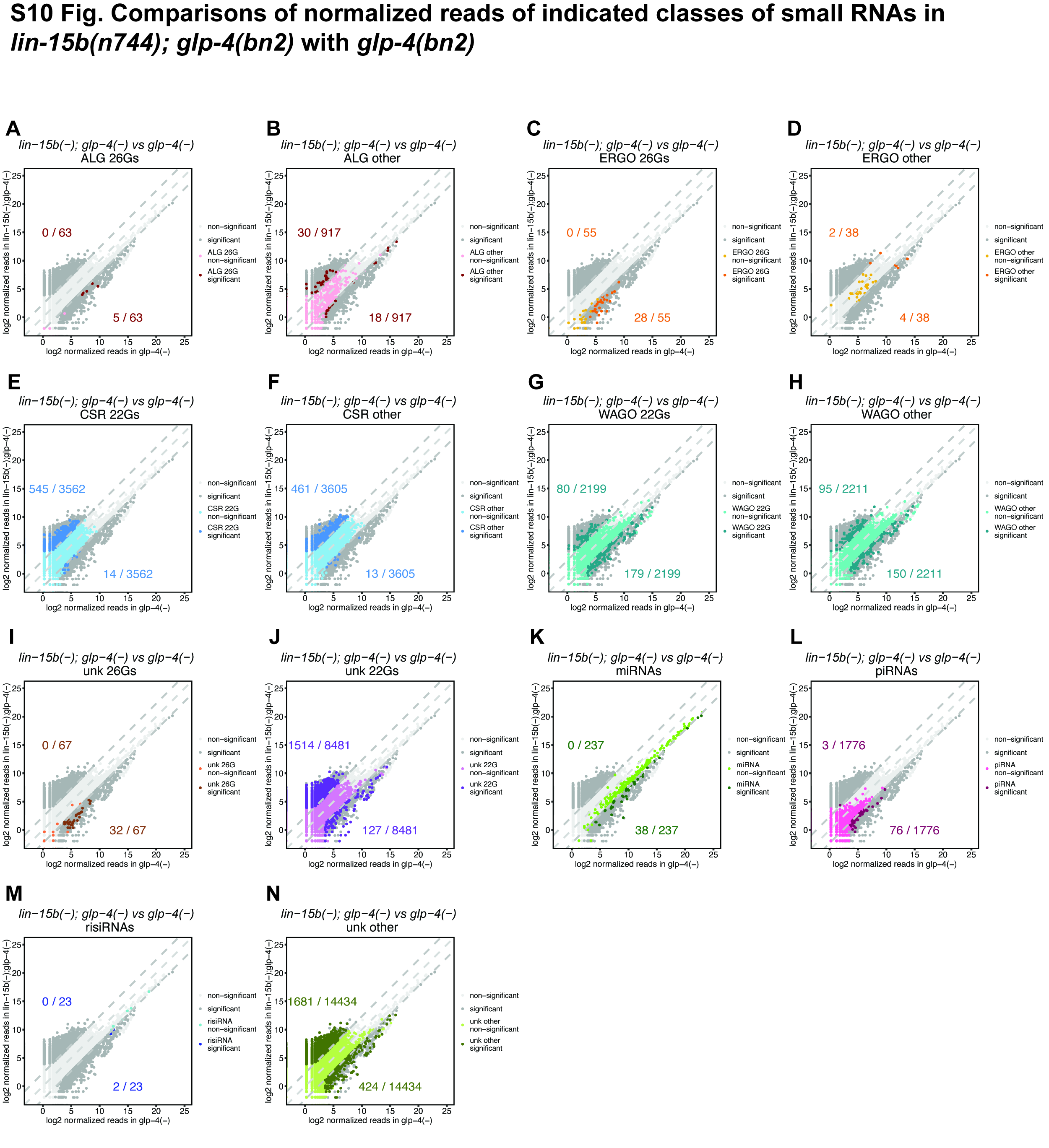

Supplement: S10 Fig — Dark gray data points represent small RNAs that are differentially expressed by 5-fold and adjusted p-value < 0.05. Colored data points represent indicated classes of small RNAs. The rest of the small RNAs are represented as light gray data points. Lines denoting equal, 5-fold increased and 5-fold decreased expression are shown as light gray dashed lines. The numbers indicate the number of small RNAs upregulated in lin-15b(n744); glp-4(bn2) (upper) and glp-4(bn2) (lower). The numerical data underlying the graphs shown in this figure can be found in the Supporting information data table named S1 Raw Data. (TIF) [file pbio.3002748.s010.tif]

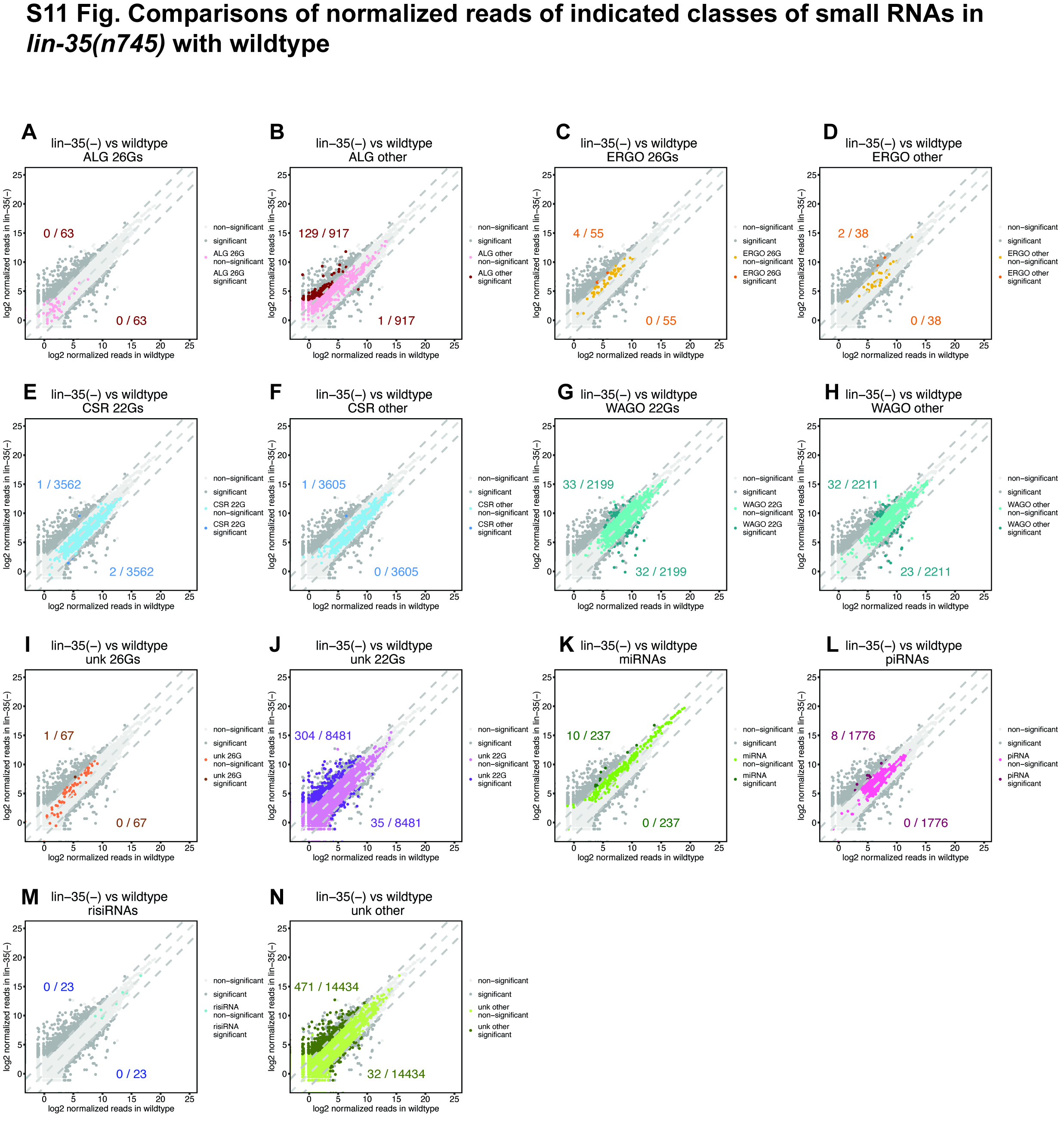

Supplement: S11 Fig — Dark gray data points represent small RNAs that are differentially expressed by 5-fold and adjusted p-value < 0.05. Colored data points represent indicated classes of small RNAs. The rest of the small RNAs are represented as light gray data points. Lines denoting equal, 5-fold increased and 5-fold decreased expression are shown as light gray dashed lines. The numbers indicate the number of small RNAs up-regulated in lin-35(n745) (upper) and wild type (lower). The numerical data underlying the graphs shown in this figure can be found in the Supporting information data table named S1 Raw Data. (TIF) [file pbio.3002748.s011.tif]

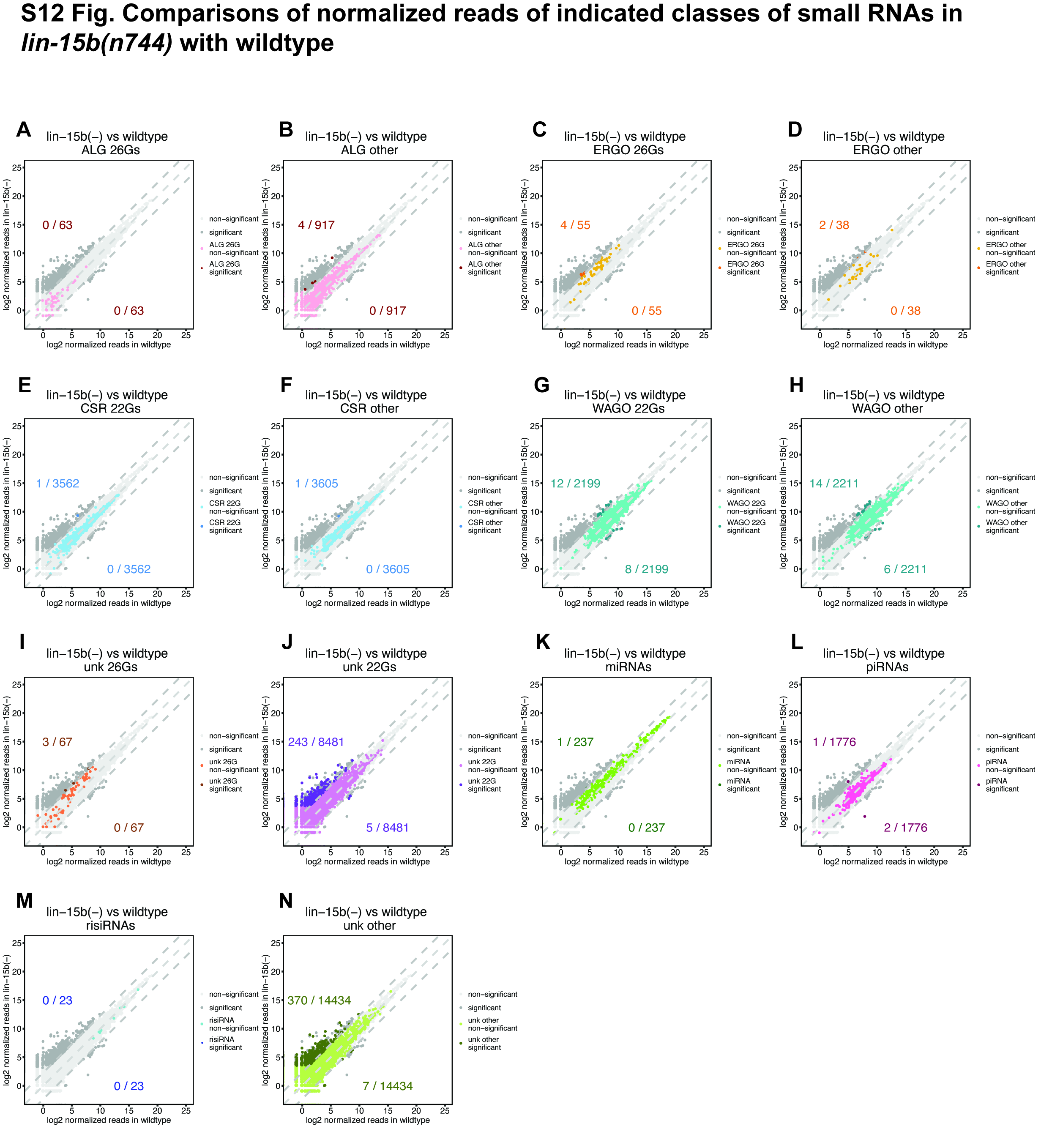

Supplement: S12 Fig — Dark gray data points represent small RNAs that are differentially expressed by 5-fold and adjusted p-value < 0.05. Colored data points represent indicated classes of small RNAs. The rest of the small RNAs are represented as light gray data points. Lines denoting equal, 5-fold increased and 5-fold decreased expression are shown as light gray dashed lines. The numbers indicate the number of small RNAs up-regulated in lin-15b(n744) (upper) and wild type (lower). The numerical data underlying the graphs shown in this figure can be found in the Supporting information data table named S1 Raw Data. (TIF) [file pbio.3002748.s012.tif]

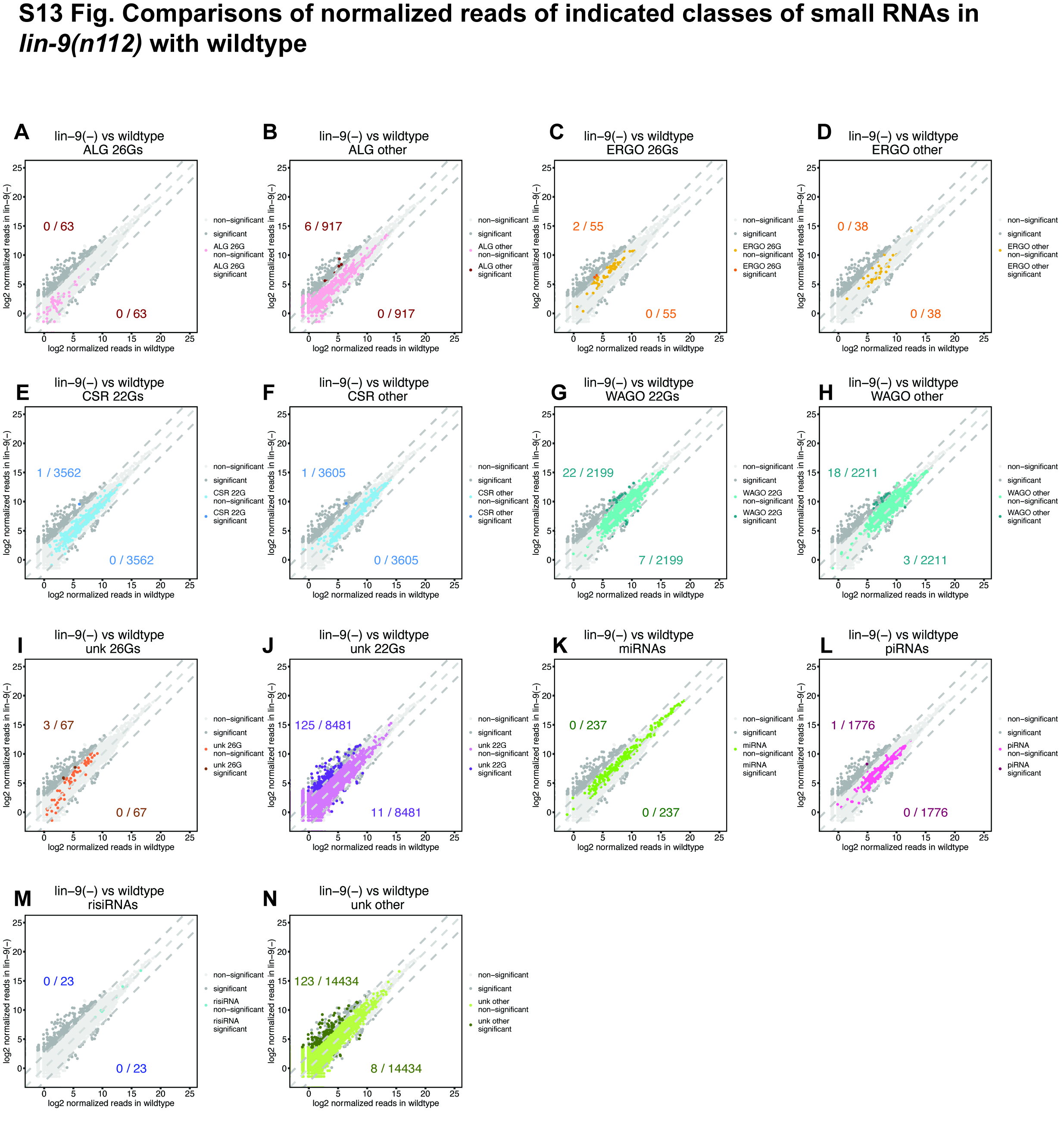

Supplement: S13 Fig — B Dark gray data points represent small RNAs that are differentially expressed by 5-fold and adjusted p-value < 0.05. Colored data points represent indicated classes of small RNAs. The rest of the small RNAs are represented as light gray data points. Lines denoting equal, 5-fold increased and 5-fold decreased expression are shown as light gray dashed lines. The numbers indicate the number of small RNAs up-regulated in lin-9(n112) (upper) and wild type (lower). The numerical data underlying the graphs shown in this figure can be found in the Supporting information data table named S1 Raw Data. (TIF) [file pbio.3002748.s013.tif]

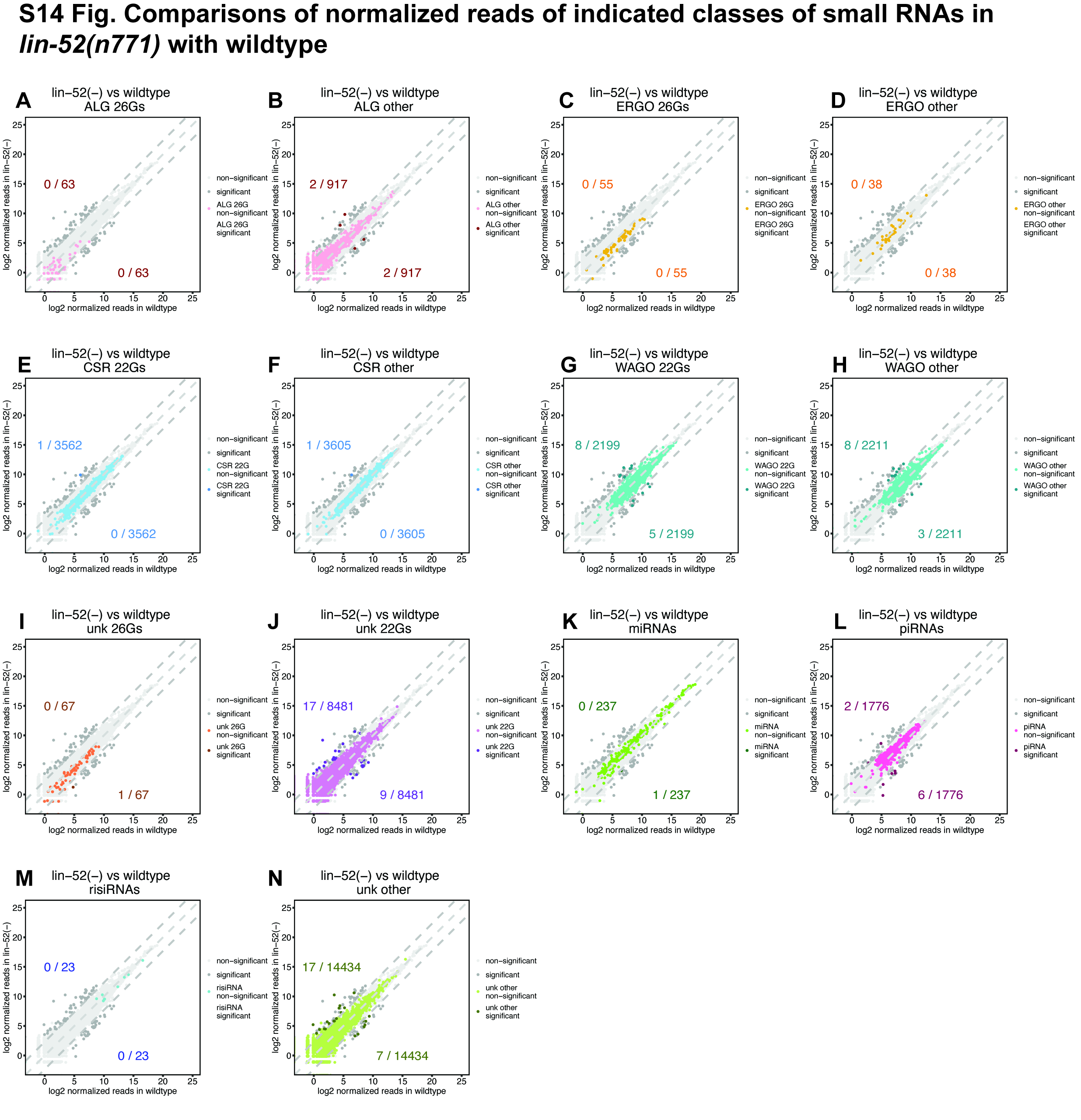

Supplement: S14 Fig — Dark gray data points represent small RNAs that are differentially expressed by 5-fold and adjusted p-value < 0.05. Colored data points represent indicated classes of small RNAs. The rest of the small RNAs are represented as light gray data points. Lines denoting equal, 5-fold increased and 5-fold decreased expression are shown as light gray dashed lines. The numbers indicate the number of small RNAs up-regulated in lin-52(n771)(upper) and wild type (lower). The numerical data underlying the graphs shown in this figure can be found in the Supporting information data table named S1 Raw Data. (TIF) [file pbio.3002748.s014.tif]
